# Wnt signaling and Loxl2 promote aggressive osteosarcoma

**DOI:** 10.1038/s41422-020-0370-1

**Published:** 2020-07-20

**Authors:** Kazuhiko Matsuoka, Latifa Bakiri, Lena I. Wolff, Markus Linder, Amanda Mikels-Vigdal, Ana Patiño-García, Fernando Lecanda, Christine Hartmann, Maria Sibilia, Erwin F. Wagner

**Affiliations:** 1 Laboratory Genes and Disease, Department of Dermatology, Medical University of Vienna (MUV), Vienna, 1090 Austria; 2 Laboratory Genes and Disease, Department of Laboratory Medicine, Medical University of Vienna (MUV), Vienna, 1090 Austria; 3 Genes, Development and Disease Group, Spanish National Cancer Research Centre (CNIO), Madrid, 28029 Spain; 4 Department of Bone and Skeletal Research, Medical Faculty, Institute of Musculoskeletal Medicine, University of Münster, Münster, 48149 Germany; 5 Department of Medicine I, Comprehensive Cancer Center, Institute of Cancer Research, Medical University of Vienna (MUV), Vienna, 1090 Austria; 6 Gilead Sciences Inc., Foster City, CA 94404 USA; 7 Navarra Institute for Health Research(IdISNA) and Program in Solid Tumors, Center for Applied Medical Research (CIMA), University of Navarra, Pamplona, 31008 Spain; 8 Department of Pediatrics, University Clinic of Navarra, Pamplona, 31008 Spain; 9 Centro de Investigación Biomédica en Red de Cáncer (CIBERONC), Pamplona, 31008 Spain

**Keywords:** Bone cancer, Mechanisms of disease

## Abstract

Osteosarcoma (OS) is the most frequent primary malignant bone tumor in urgent need of better therapies. Using genetically modified mouse models (GEMMs), we demonstrate that Wnt signaling promotes c-Fos-induced OS formation via the actions of the collagen-modifying enzyme Loxl2. c-Fos/AP-1 directly regulates the expression of the Wnt ligands *Wnt7b* and *Wnt9a* in OS cells through promoter binding, and Wnt7b and Wnt9a in turn promote Loxl2 expression in murine and human OS cells through the transcription factors Zeb1 and Zeb2. Concordantly, inhibition of Wnt ligand secretion by inactivating the *Wnt-less* (*Wls)* gene in osteoblasts in c-Fos GEMMs either early or in a therapeutic setting reduces Loxl2 expression and progression of OS. Wls-deficient osteosarcomas proliferate less, are less mineralized and are enriched in fibroblastic cells surrounded by collagen fibers. Importantly, Loxl2 inhibition using either the pan-Lox inhibitor BAPN or a specific inducible shRNA reduces OS cell proliferation in vitro and decreases tumor growth and lung colonization in murine and human orthotopic OS transplantation models. Finally, OS development is delayed in c-Fos GEMMs treated with BAPN or with specific Loxl2 blocking antibodies. Congruently, a strong correlation between c-FOS, LOXL2 and WNT7B/WNT9A expression is observed in human OS samples, and c-FOS/LOXL2 co-expression correlates with OS aggressiveness and decreased patient survival. Therefore, therapeutic targeting of Wnt and/or Loxl2 should be considered to potentiate the inadequate current treatments for pediatric, recurrent, and metastatic OS.

## Introduction

Osteosarcoma (OS) is the most common primary malignant tumor of the bone. With a worldwide yearly incidence of ~1–3 cases per million, OS arises mostly in children and adolescents during bone growth,^1^ with a second incidence peak after 50 years.^2^ A potential link between OS initiation/progression and bone growth/turnover has been suggested^3^ but additional risk factors, such as aging, gender and gene mutations have been associated with OS. The current standard of care for OS includes surgery and neo-adjuvant and post-operative adjuvant chemotherapy.^4^ However, the 5-year overall survival for patients with metastatic and/or relapsed OS is ~20% and has remained virtually unchanged over the past 30 years,^5,6^ due to the still limited understanding of the molecular mechanisms of the disease.^7^

OS is composed of malignant bone-producing osteoblastic cells and mainly affect femur, tibia and humerus. There are no established molecular markers to discriminate between normal and transformed osteoblasts. OS exhibit highly heterogeneous histological characteristics ranging from low- to high-grade with the presence of osteoblastic, chondroblastic and fibroblastic components in various proportions.^8,9^ Interestingly, while overall survival was not significantly different, patients with fibroblast-rich tumors appear to respond better to pre-operative chemotherapy compared to patients with osteoblast or chondroblast-rich tumors.^10–12^ The molecular determinants of the inter- and intra-tumoral histological diversity of OS remains to be identified.^13,14^

The proto-oncogene *c-fos* is the cellular homolog of the *v-fos* oncogene present in the FBJ- and FBR-murine sarcoma viruses.^15^ c-Fos is a component of the Activator Protein-1 (AP-1) transcription factor complex, which is composed of dimers of Jun (c-Jun, JunB, JunD) and Fos (c-Fos, FosB, Fra1, Fra2) proteins.^16^ AP-1 is activated by various physiological and pathological signals such as growth factors, inflammatory cytokines, UV radiation and oxidative stress.^17–20^ Rearrangements in the *FOS* gene were recently reported in human osteoblastoma and osteoma cases^21^ and c-Fos mRNA and protein expression are elevated in human OS and in tumor cells derived from OS mouse models.^22–24^ Expression of c-Fos in H2-*c-fos*LTR transgenic mice results in OS development,^25^ and although the exogenous *c-fos* gene is expressed from the broadly active MHC Class I gene promoter, tumors arise exclusively and with 100% penetrance in bone. c-Fos-induced OS requires EGFR signaling and its downstream kinase RSK2.^26,27^ Importantly, H2-*c-fos*LTR mice exhibit numerous hallmarks of the human disease, such as early-onset tumor development in long bones and heterogeneous histology.^25,28,29^

Wnt comprises a large family of secreted proteins that bind highly conserved cell membrane receptors and co-receptors.^30^ Signaling pathways downstream of Wnt ligands are also evolutionally conserved and are critical in embryogenesis, postnatal development, pathogenesis and tumorigenesis.^30–33^ Wnt signaling can be classified as canonical or non-canonical, depending on the involvement of β-catenin as a downstream effector, although some Wnt ligands can activate both pathways.^34^ Activation of the canonical pathway causes β-catenin cytoplasmic accumulation and nuclear translocation, where it co-activates TCF/LEF transcription factors, promoting the expression of Wnt-responsive genes including Axin2, c-Myc, cyclin D1 and matrix metalloproteinases.^31^ Non-canonical Wnt signaling involves several pathways and downstream effectors, such as the planar cell polarity pathway, the Wnt/Ca^2+^ pathway and the JNK, ERK and CaMKII kinases, and it can also suppress the β-catenin pathway.^35^

Mutations in Wnt pathway genes such as WNT1 (ligand), LRP5 (co-receptor) and SOST (antagonist) have been associated with altered bone mass^36^ and SOST/Sclerostin blocking antibodies are in clinical trials for osteoporosis.^37^ Inherited inactivating mutations in Adenomatous Polyposis Coli (*APC*), a negative regulator of Wnt/β-catenin, cause Familial Adenomatous Polyposis (FAP). Osteomas are part of the extra-colonic manifestations of FAP,^38^ while APC somatic mutations are found in several tumor types including OS.^39,40^ Furthermore, Wnt target genes, such as *MYC* and *TWIST*, are amplified in human OS, while Wnt inhibitory factor 1 (*WIF1*) is epigenetically silenced.^41–43^ *WLS/GRP177*, encoding the exclusive cargo protein for Wnt ligands and essential to the secretion of all mammalian Wnts,^44,45^ was recently identified as a likely oncogene in human OS.^46^ These observations and the aberrant expression of numerous Wnt ligands, FZD/LRP receptors and Wnt inhibitors collectively support an implication of Wnt signaling in OS.

To explore a possible genetic interaction between Wnt signaling and c-Fos/AP-1 in OS and to evaluate the therapeutic benefit of targeting Wnt in OS, we genetically inactivated the mouse homolog of *WLS/GRP177* in the H2-*c-fos*LTR preclinical model for OS to inhibit Wnt ligands secretion from osteoblast progenitors and OS cells. When *Wls* was inactivated, either at the early phases of OS formation or in a therapeutic setting, OS formation was reduced and fibroblastic characteristics were favored. We further identify *Wnt7b* and *Wnt9a*, encoding two Wnt ligands, as two novel Fos/AP-1 target genes relevant for OS and the Lysyl oxidase-like2 (Loxl2) collagen-cross-linking enzyme as the important determinant of OS downstream of Wnt signaling. Importantly, LOXL2 is co-expressed with WNT7B/WNT9A and with FOS in a substantial fraction of human OS specimens and therapeutic targeting of Loxl2 with a small molecule inhibitor, with shRNA or with blocking antibodies delays OS formation in experimental OS models.

## Results

### Wntless (Wls) inactivation reduces tumor burden in vivo

An inducible gene inactivation strategy based on the Osterix (Osx) promoter and Doxycycline (Dox) was used to circumvent the effects of constitutive and embryonic *Wls* inactivation in bone cells reported with the Osteocalcin-Cre^47^ or Col1a1-Cre^48^ alleles. Dox-inducible and osteoblast-specific Wls loss-of-function OS mice were generated combining *Wls* floxed,^49^ Osx-tetO-cre^50^ and H2-*c-fos*LTR alleles^25^ by genetic crosses. In the resulting Wls^ΔOB^-OS mice, Cre expression is suppressed by Dox administration to pregnant mothers and their progenies. Wls^ΔOB^-OS mice and their control littermates carrying combinations of the different alleles are born at the expected frequencies and appeared healthy with no detectable bone abnormalities.

**Fig. 1:**
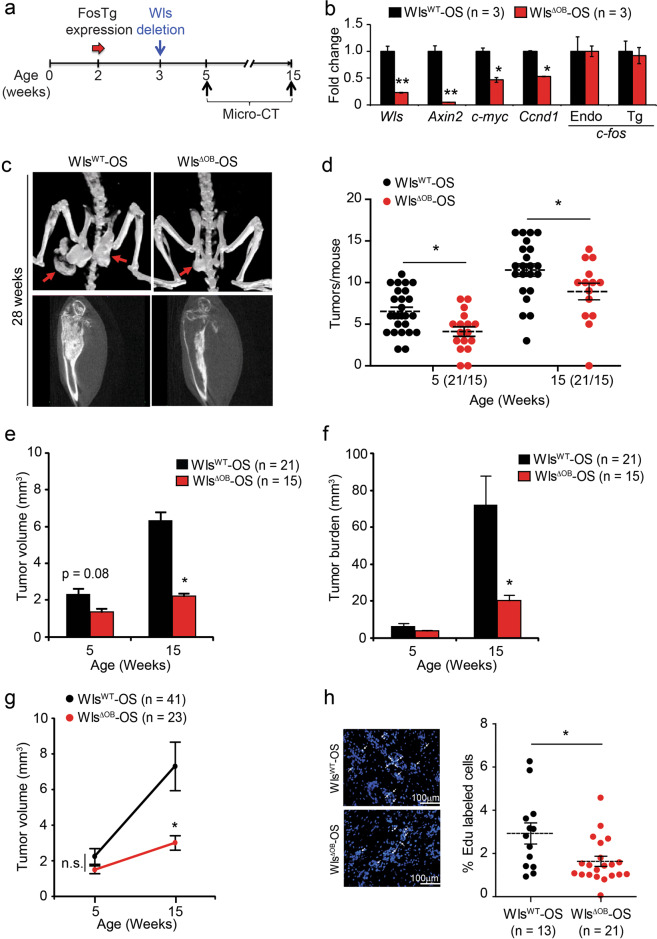
Wnt-less (Wls) deletion in osteoblast progenitors reduces c-Fos-induced OS. **a** Experimental procedure to prevent Wnt secretion by deleting the Wls gene in osteoblast progenitors. Dox is removed at the onset of tumor formation (3 weeks of age, 1 week after Fos transgene expression is detected) and tumors are monitored at 5 and 15 weeks of age. **b** qPCR analysis of *Wls*, Wnt target genes and Fos mRNA species (endo, endogenous, Tg, transgene) in Wls^WT^ and Wls^ΔOB^-OS tumor-bearing bones at 5 weeks of age. **c**–**g** Micro-CT analysis of Wls^WT^ and Wls^ΔOB^-OS mice. Representative Micro-CT: 3D reconstructions (Top) and 2D virtual sections (bottom) of tumor-bearing bones. Red arrows point to OS (**c**). Tumor number per mouse (**d**), average tumor volume (**e**) and average tumor burden per mouse (**f**) at 5 and 15 weeks in Wls^WT^-OS mice (*n* = 21) and Wls^ΔOB^-OS mice (*n* = 15). **g** Volumetric follow-up over time for 41 Wls^WT^-OS tumors and 23 Wls^ΔOB^-OS tumors. **h** EdU incorporation by Wls^WT^-OS (*n* = 13) and Wls^ΔOB^-OS (*n* = 21) tumors at 15 weeks of age. The left panels are representative images of EdU-labeled cells 4 h after EdU injection (white arrows, nuclei counterstained with DAPI) while % EdU-labeled cells per tumor are plotted on the right. Bar graphs and plots represent or include mean ± SEM, respectively. *P < 0.05, **P < 0.01.

By removing Dox at 3 weeks of age, Cre expression was induced close to the onset of OS formation in H2k-*c-fos*LTR mice^25^ and OS development was monitored by longitudinal Micro-CT (Fig. 1a). Tissue density in trabecular bone regions of wild-type/control littermates was used to compute the cut-off value to distinguish between bone/OS and non-mineralized tissues in the Micro-CT analysis. Two weeks after Dox removal, at 5 weeks of age, quantitative real-time PCR (qPCR) analyses indicated that *Wls* expression was significantly decreased in tumor-bearing bones from Wls^ΔOB^-OS mice compared to *Wls* floxed, H2k-*c-fos*LTR littermates that do not carry the Osx-tetO-cre allele (Wls^WT^-OS), consistent with activation of Cre expression (Fig. 1b). About 80% decrease in *Wls* expression was also observed in primary OS cells derived from Wls^ΔOB^-OS tumors (Supplementary information, Fig. S1a). The Wnt/β-catenin target genes *Axin2*, *c-myc* and *Ccnd1* were also significantly decreased in Wls^ΔOB^-OS tumor-bearing bones (Fig. 1b) and primary OS cells (Supplementary information, Fig. S1a), while endogenous *c-fos* and the *c-fos* transgene were not affected. Wls^WT^-OS and Wls^ΔOB^-OS mice exhibited Micro-CT-detectable OS in several bones including femur, tibia and pelvis (Fig. 1c), but Wls^ΔOB^-OS mice had significantly fewer tumors than Wls^WT^-OS mice at 5 and 15 weeks (Fig. 1d). In addition, the average volume of the tumors (Fig. 1e) and the overall tumor burden per mouse (Fig. 1f) were smaller in Wls^ΔOB^-OS mice at 15 weeks. Furthermore, when Wls was inactivated at birth before the onset of c-Fos transgene expression (Supplementary information, Fig. S1b), very few tumors were observed in 5 week-old Wls^ΔOB^-OS mice and tumor volume and burden were also decreased (Supplementary information, Fig. S1c–e). Heterozygous *Wls* inactivation at 3 weeks of age in Wls^HET^-OS mice that carry the Osx-tetO-cre allele, but only one floxed *Wls*, did not affect OS number, burden or growth (Supplementary information, Fig. S1f–h). This further excludes a possible contribution from the Osx-tet-cre allele to the observed phenotypes.

Finally, we monitored individual tumors that were of roughly similar size at 5 weeks (2 weeks after homozygous *Wls* inactivation) and observed that Wls^ΔOB^-OS tumors grew slower than Wls^WT^-OS tumors, reaching a significantly smaller size 10 weeks later (Fig. 1g). This is consistent with decreased *c-myc* and *Ccnd1* expression (Fig. 1b) and likely due to decreased proliferation as the number of EdU-labeled cells was significantly lower in Wls^ΔOB^-OS tumors (Fig. 1h). These results indicate that early *Wls* inactivation in osteoblasts/OS cells delays OS development and suggest that Wnt signaling is critical for c-Fos-induced OS growth.

### Osteoblast-specific Wls deficiency affects OS pathology

**Fig. 2:**
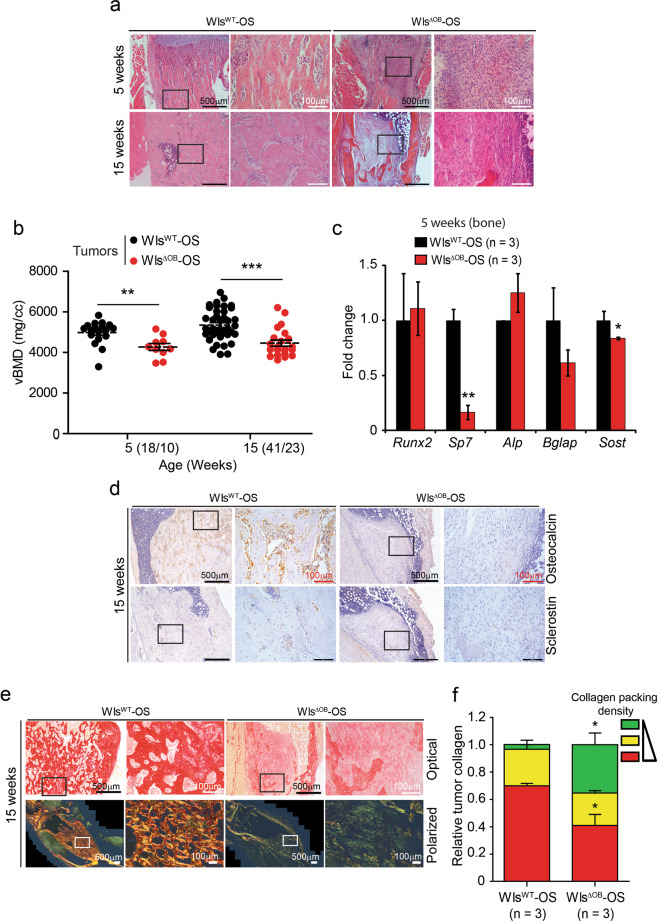
Wls deficiency in osteoblast progenitors affects OS pathology. **a** Representative H*&*E stainings of Wls^WT^-OS and Wls^ΔOB^-OS tumors at 5 and 15 weeks. **b** Micro-CT measurement of vBMD in Wls^WT^-OS and Wls^ΔOB^-OS tumors at 5 and 15 weeks of age. The total number of analyzed tumors is indicated between parentheses (WT/ΔOB) beside each time point. **c** qPCR analysis of osteoblast/osteocyte marker in tumor-bearing bones from 5 week-old Wls^WT^-OS and Wls^ΔOB^-OS mice. **d** IHC analysis of Osteocalcin and Sclerostin at 15 weeks. **e** Picrosirius red staining of Wls^WT^-OS and Wls^ΔOB^-OS tumors photographed under optical (Top) and polarized (Bottom) light. **f** Quantification of collagen packing density in the tumors using picrosirius red staining and polarized light at 15 weeks (*n* = 3). Bar graphs and plots represent or include mean ± SEM, respectively. *P < 0.05, **P < 0.01.

Wls^WT^-OS mice develop at 5 and 15 weeks osteoblastic-like OS composed of immature bone/osteoid, while tumors from Wls^ΔOB^-OS mice appeared less bony and enriched in fibrotic-like cells and extracellular matrix (ECM) (Fig. 2a). Consistently, Micro-CT analyses revealed that volumetric bone mineral density (vBMD) in the tumor was lower in Wls^ΔOB^-OS than in Wls^WT^-OS (Fig. 2b), while cortical vBMD was not changed (Supplementary information, Fig. S2a). Circulating bone formation (P1NP) and bone resorption (CTX) markers were not different between genotypes at 5 and 15 weeks (Supplementary information, Fig. S2b, c) and mRNA expression of the major collagens *Col1a1*, *Col1a2* and *Col3a1* appeared comparable in tumor-bearing bones at 5 weeks and in primary OS cells (Supplementary information, Fig. S2d, e). Consistent with unaltered CTX, mRNA expression of important modulators of osteoclastogenesis *Tnfrs11b*, *Tnfsf11* and *Csf1*, encoding for Opg, Rankl and M-Csf, and the expression of the osteoclast markers Acp5, Itgb3 and Oscar were unaffected (Supplementary information, Fig. S2f). mRNA expression of the osteo-chondro progenitor transcription factor *Runx2* and the intermediate osteoblast marker *Alp* were also not significantly changed (Fig. 2c and Supplementary information, Fig. S2g). Importantly, the osteoblast transcription factor *Sp7*, encoding Osx, and the late-osteoblast/osteocyte maker *Sost*, encoding Sclerostin, were significantly decreased in tumor-bearing bones from Wls^ΔOB^-OS mice, while the decrease in *Bglap* mRNA, encoding the mature osteoblast marker Osteocalcin, did not reach statistical significance (Fig. 2c). qPCR analyses in primary OS cells and Cre immunohistochemistry (IHC) used as a reporter for *Sp7/*Osx expression by comparing Wls^HET^-OS and Wls^ΔOB^-OS tumors, confirmed reduced transcription of *Sp7*, when Wls is inactivated (Supplementary information, S2g–i). Finally, Osteocalcin and Sclerostin IHC further documented a striking decrease in Osteocalcin- and Sclerostin-positive cells in Wls^ΔOB^-OS tumors at 15 weeks (Fig. 2d).

Collagen networks in the tumor were next assessed by picrosirius red staining and visualization under optical or polarized light. At 15 weeks, tumors from Wls^WT^-OS mice were mostly composed of type I collagen with tightly packed red/orange bundles under polarized light, while collagens in the Wls^ΔOB^-OS fibrotic fibers appeared less stained and of a greenish color consistent with reduced packing (Fig. 2e). Quantification of these images further documented the overall decrease in collagen packing in Wls^ΔOB^-OS tumors (Fig. 2f). These data indicate that Wls^ΔOB^-OS tumors present histological and molecular features of fibroblastic OS with decreased mineralization and a notable change in collagen fiber organization.

### Wls inactivation in a therapeutic setting inhibits OS growth

**Fig. 3:**
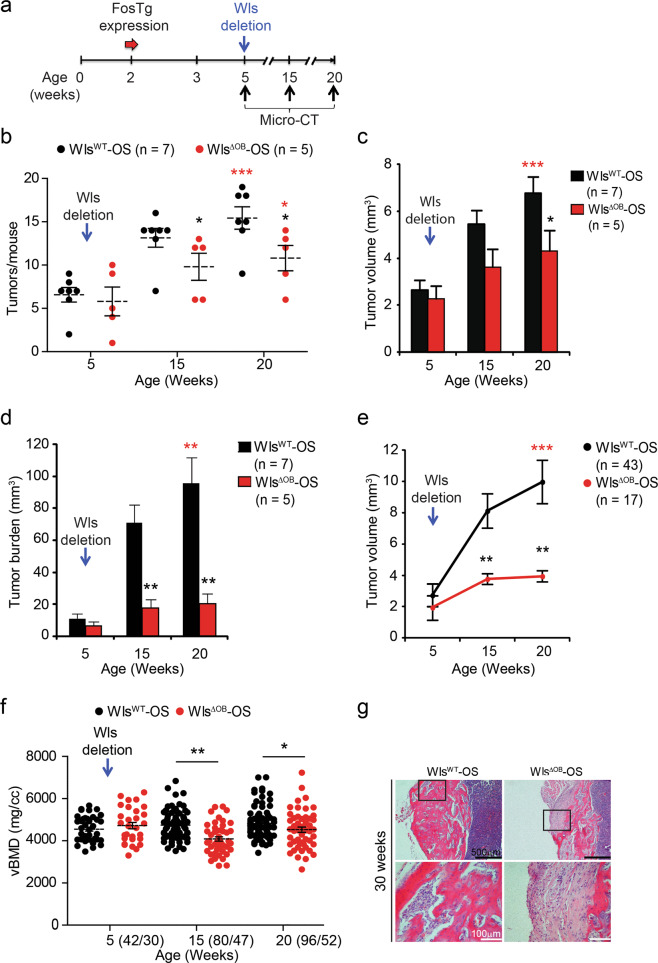
Wls deletion during tumor progression decreases OS in H2-*c-fos*LTR mice. **a** Experimental procedure. Dox is removed at 5 weeks of age when tumors are detectable by Micro-CT and the mice are additionally subjected to Micro-CT at 15 and 20 weeks. **b**–**f** Micro-CT analysis of Wls^WT^ and Wls^ΔOB^-OS mice. Tumor number per mouse (**b**), average tumor volume (**c**) and average tumor burden per mouse (**d**) at 5, 15 and 20 weeks in Wls^WT^-OS mice (*n* = 7) and Wls^ΔOB^-OS mice (*n* = 5). **e** Volumetric follow-up over time for 43 Wls^WT^-OS tumors and 17 Wls^ΔOB^-OS tumors. **f** vBMD in Wls^WT^-OS and Wls^ΔOB^-OS tumors over time. The number of analyzed tumors is indicated in parentheses (WT/ΔOB) beside each time point. **g** Representative tumor histology at 30 weeks. Bar graphs and plots represent or include mean ± SEM, respectively. Black asterisks: *P < 0.05 and **P < 0.01 by two-way ANOVA with Bonferroni post hoc test. Red asterisks: ns not significant, *P < 0.05, **P < 0.01 and ***P < 0.001 by two-tailed *t*-test between each genotype at 20 weeks and all mice at 5 weeks.

The effect of Wls inactivation in established OS was assessed next by removing Dox at 5 weeks of age (Fig. 3a). Micro-CT analyses confirmed that tumor number, size and burden were indistinguishable between genotypes at the 5-week time point (Fig. 3b–d). However, the three parameters were substantially lower in Wls^ΔOB^-OS than in Wls^WT^-OS mice, when measured 10 and 15 weeks later (Fig. 3b–d). Notably, the increase in tumor size and tumor burden between Wls inactivation (5 weeks) and end-point (20 weeks) was only significant in Wls^WT^-OS mice (Fig. 3c, d). Individual tumor follow-up further indicated that Wls^ΔOB^-OS tumors not only grew slower than Wls^WT^-OS controls, but did not significantly increase in size relative to the 5-week starting point (Fig. 3e). Remarkably, growth kinetics of Wls^ΔOB^-OS tumors was substantially delayed with 70% decrease in tumor volume at 20 weeks. Micro-CT quantification over time and histological analyses at the end-point indicated that Wls^ΔOB^-OS tumors had consistently lower vBMD (Fig. 3f) and displayed fibroblastoid characteristics (Fig. 3g), similar to what was observed when Wls was inactivated at the onset of OS development. These data indicate that Wnt signaling is essential to sustain c-Fos-induced OS growth and targeting Wnt signaling is a valuable therapeutic option in this preclinical OS model.

### *Wnt7b* and *Wnt9a* are novel c-Fos/AP-1 transcriptional targets in OS

**Fig. 4:**
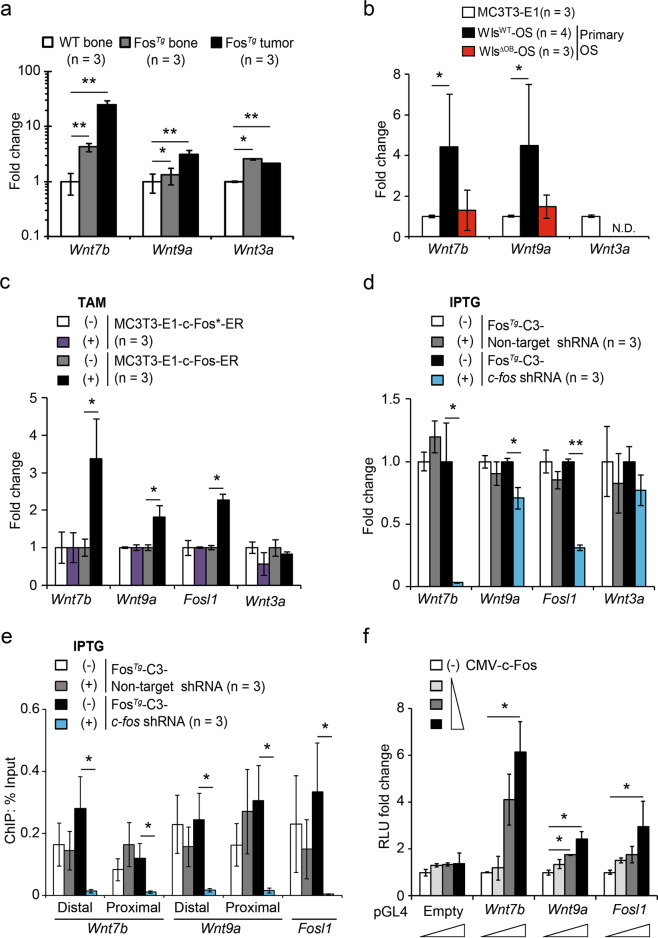
c-Fos/AP-1 directly promotes the expression of Wnt7b and Wnt9a in OS. **a**, **b** qPCR analysis of *Wnt7b*, *Wnt9a* and *Wnt3a* in H2-*c-fos*LTR bones, tumor-bearing bones and dissected tumors (**a**) and primary OS cells isolated from Wls^WT^-OS and Wls^ΔOB^-OS mice (**b**). Bones from WT littermates (**a**) and MC3T3-E1 osteoblastic cells (**b**) were included for comparison. N.D., not detectable. **c**, **d** Gene expression in MC3T3-E1 cells expressing c-Fos-ER or mutant (inactive) c-Fos*-ER in the presence/absence of tamoxifen (**c**) and in the H2-*c-fos*LTR OS cell line (Fos^Tg^-C3) expressing IPTG-inducible *c-fos* shRNA or non-target shRNA in the presence/absence of IPTG (**d**) was determined by qPCR. *FosL1* (encoding Fra-1 and a bona fide Fos-target gene) is included as a control. **e** ChIP-qPCR quantification of c-Fos/AP-1 binding to *Wnt7b* and *Wnt9a* promoters in H2-*c-fos*LTR OS cells expressing IPTG-inducible *c-fos* shRNA or non-target shRNA in the presence/absence of IPTG. The *FosL1* genomic region with a c-Fos/AP-1 binding site is included as a control. **f** Luciferase reporter assay in MC3T3-E1 cells cotransfected with increased amounts of c-Fos expressing vector (CMV-c-Fos) and *Wnt7b* and *Wnt9a* reporter constructs containing the ChIP sites from **e**. A Fosl1 reporter construct is included as a control. Bar graphs represent mean ± SEM. *P < 0.05 and **P < 0.01.

Screening for Wnt ligand expression by qPCR revealed that *Wnt7b*, *Wnt9a* and to a lesser extent *Wnt3a*, were significantly elevated in H2-*c-fos*LTR tumor-bearing bones and tumors (Fig. 4a). *Wnt7b* and *Wnt9a*, but not *Wnt3a*, mRNA expression was also specifically increased in cultured primary OS cells isolated from Wls^WT^-OS tumors, when compared to MC3T3-E1 osteoblastic cells and, although not statistically significant, to primary OS isolated Wls^ΔOB^-OS mice (Fig. 4b), indicating that c-Fos might positively control *Wnt7b* and *Wnt9a* expression. In contrast, while most Wnt receptors and co-receptor mRNAs were readily detectable in control, H2-*c-fos*LTR tumor-bearing bones, tumors, primary and OS cell lines, no consistent correlation with c-Fos expression was observed (Supplementary information, Fig. S3a, b).

We next examined the regulation of *Wnt7b* and *Wnt9a* by c-Fos. MC3T3-E1 osteoblastic cell lines expressing tamoxifen-regulatable Fos-ER or DNA-binding-deficient Fos*-ER fusion proteins^51^ were established (Supplementary information, Fig. S3c). Forty-eight hours after tamoxifen stimulation, mRNA expression of *Wnt7b, Wnt9a* and *Fosl1*, a bona fide Fos transcriptional target, increased in MC3T3-Fos-ER cells, but not in MC3T3-Fos*-ER cells (Fig. 4c). Fos^Tg^-C3-c-Fos shRNA cells were derived from a previously established H2-*c-fos*LTR OS cell line^25^ to express an IPTG-inducible c-Fos shRNA (Supplementary information, Fig. S3d). Mirroring the ectopic expression data, IPTG treatment resulted in a marked decrease of *Wnt7b, Wnt9a* and *Fosl1* mRNA expression in Fos^Tg^-C3-c-Fos shRNA cells, but not in Fos^Tg^-C3-cells expressing a non-targeting shRNA (Fig. 4d). Analyses of the murine *Wnt7b* and *Wnt9a* promoter regions revealed 2 putative AP-1-binding elements (Supplementary information, Fig. S3e). c-Fos chromatin immunoprecipitation (ChIP) comparing IPTG-treated Fos^Tg^-C3-c-fos shRNA cells to untreated cells and to Fos^Tg^-C3-non-target shRNA indicated efficient c-Fos/AP-1 binding to chromatin fragments of the *Wnt7b* and *Wnt9a* promoters containing the putative sites only under conditions when Fos was expressed (Fig. 4e). Furthermore, c-Fos activated *Wnt7b* and *Wnt9a* or *Fosl1* luciferase reporters in a dose-dependent manner upon transient co-transfection in MC3T3-E1 cells (Fig. 4f). These data indicate that c-Fos-containing AP-1 dimers directly and positively control *Wnt7b* and *Wnt9a* transcription in mouse OS and osteoblastic cells.

Finally, FOS was knocked-down using Dox-inducible shRNA in human LM7 (Supplementary information, Fig. S4a) and 143b OS cell lines.^27^ Decreased mRNA expression of WNT7B and, to a lesser extent, WNT9A was observed when FOS was knocked down (Supplementary information, Fig. S4b, d). Conversely, transient FOS over-expression increased WNT7B and WNT9A expression in two human OS cell lines (Supplementary information, Fig. S4c, e), indicating that c-Fos also regulates WNT7B and WNT9A expression in human OS.

### Lysyl oxidase-like 2 is a downstream Wnt target essential for OS proliferation

**Fig. 5:**
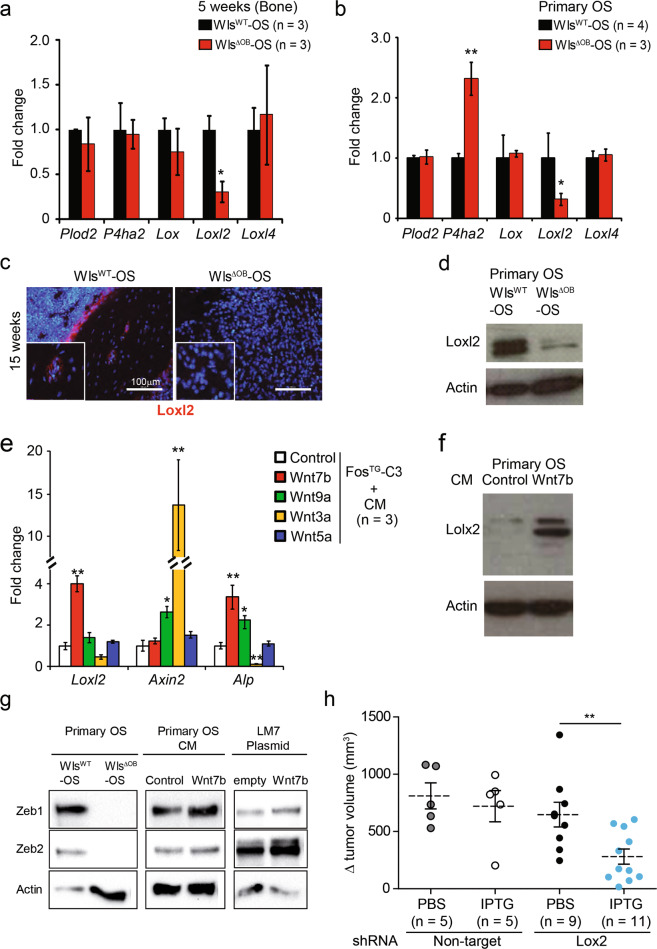
Loxl2 expression is modulated by Wnt and is essential for OS proliferation. **a**, **b** qPCR analysis of collagen modifying enzymes in tumor-bearing bone (**a**) and primary OS cells (**b**) isolated from Wls^WT^-OS and Wls^ΔOB^-OS mice. **c** Loxl2 immunofluorescence (red) in Wls^ΔOB^-OS and Wls^WT^-OS tumors at 15 weeks. Nuclei are counterstained with DAPI. **d** Loxl2 immunoblot in primary tumor cells isolated from Wls^WT^-OS and Wls^ΔOB^-OS mice. **e** qPCR analysis of *Loxl2* and the Wnt targets *Axin2* and *Alp* in Fos^Tg^-C3 cells stimulated with Wnt7b, Wnt9a, Wnt3a, Wnt5a or control conditioned medium (CM) for 48 h. **f** Loxl2 immunoblot in primary tumor cells isolated from H2-*c-fos*LTR OS cells treated with control or Wnt7b-CM for 48 h. **g** Immunoblot for Zeb1 and Zeb2 in in primary tumor cells isolated from Wls^WT^-OS and Wls^ΔOB^-OS mice, in Fos^Tg^-C3 cells treated with Wnt7b CM and in LM7 cells ectopically expressing WNT7B. **h** Bone tumor burden 21 days post cell implantation in NSG mice orthotopically injected with Fos^Tg^-C3 cells expressing IPTG-inducible *Loxl2* shRNA or non-target shRNA and treated with IPTG or vehicle (PBS) during 18 days. Actin is used as a loading control for immunoblot. Bar graphs and plots represent or include mean ± SEM, respectively. *P < 0.05 and **P < 0.01.

We next investigated the molecular basis of altered collagen fiber organization observed in the tumor of Wls^ΔOB^-OS mice. Post-translational modifications are critical to the structure and biological function of collagen.^52^ Since collagen expression and collagen-related serum parameters such as P1NP and CTX were not changed between Wls^WT^-OS and Wls^ΔOB^-OS mice, we analyzed the expression of a panel of collagen-modifying genes. Among collagen cross-linking enzymes, Lysyl oxidase-like 2 (Loxl2) mRNA was significantly and specifically decreased in Wls^ΔOB^-OS tumor-bearing bones and primary OS cells (Fig. 5a, b). Decreased Loxl2 was also observed in Wls^ΔOB^-OS bone tumors by IHC, regardless whether *wls* was inactivated preventively (at 3 weeks; Fig. 5c) or therapeutically (at 5 weeks; Supplementary information, Fig. S5a). Loxl2 was also found decreased in Wls^ΔOB^-OS primary OS cells by immunoblot analyses (Fig. 5d), suggesting that Wnt signaling is an important regulator of Loxl2 expression and collagen cross-linking activity in OS.

Conditioned medium (CM) was next prepared from L cells expressing Wnt3a, Wnt5a, Wnt7b or Wnt9a and used to stimulate Fos^Tg^-C3 OS cells in vitro. As shown in Fig. 5e, f, Loxl2 expression was specifically induced by Wnt7b-CM and its mRNA induction is comparable to the Wnt7b target *Alp*, encoding Alkaline phosphatase^53^ (Fig. 5e). *Alp* was also induced by Wnt9a-CM while the canonical Wnt/Δ-catenin target *Axin2* was robustly induced by Wnt3a-CM and only modestly by Wnt9a-CM (Fig. 5e). Importantly, Loxl2 mRNA expression was increased in Fos^Tg^-C3 cells when stimulated with recombinant Wnt9a (Supplementary information, Fig. S5b) and in MC3T3-E1 osteoblastic cells transiently transfected with Wnt7b or Wnt9a expression vectors (Supplementary information, Fig. S5c), while the prototypic canonical (Wnt3a) or non-canonical (Wnt5a) Wnt ligands had either no (Fig. 5e) or a repressive (Supplementary information, Fig. S5c) effect on Loxl2 mRNA. Increased Loxl2 protein expression was also observed in primary or established OS cell lines derived from Fos^Tg^ mice transfected with Wnt7b or Wnt9a expression vectors (Supplementary information, Fig. S5d). LOXL2 mRNA and protein expression were also increased in human OS cell lines transfected with WNT7B or WNT9A vectors (Supplementary information, Fig. S5e, f). Finally, Zeb1, a transcription factor reported to directly control Loxl2 expression^54^ and its close homolog Zeb2, were stimulated by Wnt7b in mouse and human OS cells (Fig. 5g), while *Wls* was essential for Zeb1 and Zeb2 expression in primary mouse OS cells (Fig. 5g) and in OS sections (Supplementary information, Fig. S5g). Collectively, these data demonstrate that Wnt7b and Wnt9a modulate Loxl2 expression in murine and human cells possibly through Zeb1/2.

We next assessed the functional relevance of Loxl2 expression in OS. IPTG-inducible shRNA knockdown of Loxl2 (Supplementary information, Fig. S6a) substantially decreased the proliferation of Fos^Tg^-C3 cells in vitro (Supplementary information, Fig. S6b). Furthermore, immune-deficient mice injected intratibially with Fos^Tg^-C3 cells expressing IPTG inducible shRNA-Loxl2 and subsequently treated with IPTG (Supplementary information, Fig. S6c) developed significantly smaller bone tumors compared to PBS-treated mice or to mice injected with Fos^Tg^-C3 cells expressing non-target shRNA (Fig. 5h; Supplementary information, Fig. S6d). Decreased Loxl2 expression and collagen packing were specifically observed in tumors from mice injected with Fos^Tg^-C3-shRNA-Loxl2 cells and treated with IPTG (Supplementary information, Fig. S6e, f). Interestingly, macroscopic lung colonization was not visible in these mice (Supplementary information, Fig. S6g), while all other groups developed multiple lung nodules, as previously reported in this orthotopic OS model.^55^ These data indicate that Loxl2 is an important functional determinant of OS development downstream of Fos and Wnt signaling.

### Lysyl oxidase or Loxl2 inhibition reduces OS in vivo

**Fig. 6:**
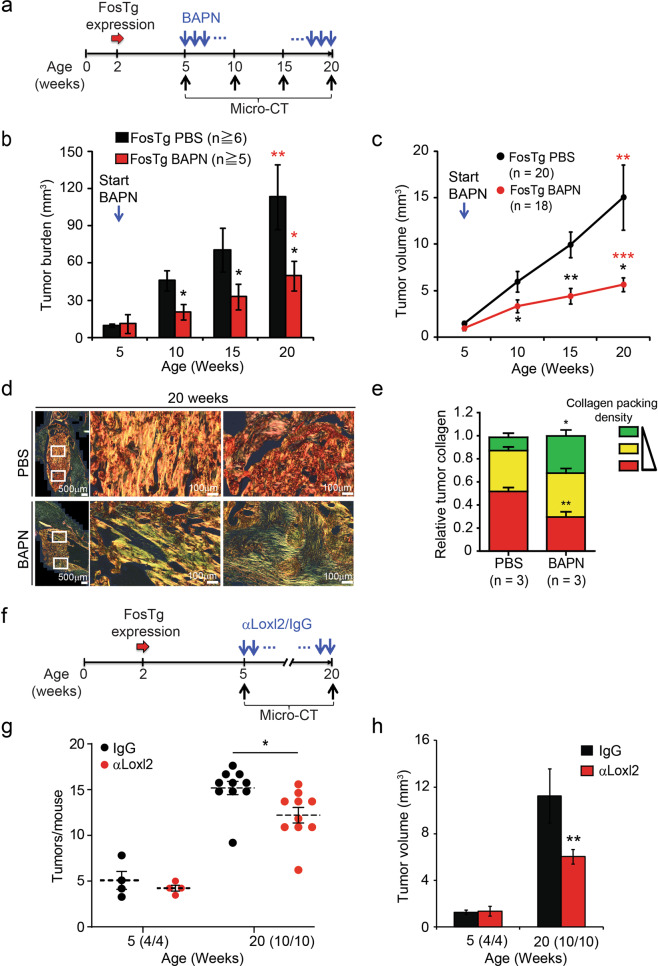
Inhibition of Lysyl oxidase activity or Loxl2 reduces OS growth. **a** Experimental procedure: 5-week-old H2-*c-fos*LTR mice were injected three times/week during 15 weeks with 250 mg/kg BAPN to block lysyl oxidase activity or with vehicle (PBS) and tumors were monitored longitudinally by Micro-CT. **b** Average tumor burden per mouse. **c** Volumetric follow-up over time for 20 and 18 tumors from PBS-treated and BAPN-treated mice, respectively at 5, 10, 15 and 20 weeks in PBS- or BAPN-treated mice. **d**, **e** Representative images of Picrosirius red/polarized light staining (**d**) and quantification of tumor collagen packing density **(e)** from tumor-bearing PBS- and BAPN-treated mice at 20 weeks. **f**–**h** Experimental procedure: 5-week old H2-*c-fos*LTR mice were injected two times/week during 15 weeks with anti-Loxl2 blocking antibody (30 mg/kg) or with IgG and tumors were monitored longitudinally by Micro-CT (**f**) for tumor number per mouse **(g)** and average tumor volume **(h)** at 5 and 20 weeks. Bar graphs and plots represent or include mean ± SEM, respectively. Black asterisks: *P < 0.05 and **P < 0.01 by two-way ANOVA with Bonferroni post hoc test. Red asterisks: *P < 0.05, **P < 0.01 and ***P < 0.001 by two-tailed *t*-test between PBS- and BAPN-treated at end point and all mice at start.

We next assessed the relevance of targeting Loxl2 and more generally lysl oxidases in OS using small inhibitory compounds. The pan-lysyl oxidase inhibitor, beta-aminopropionitrile (BAPN), is an irreversible, active site-directed inhibitor of the Lox/Loxl family.^56,57^ In line with our observations using Loxl2 shRNA knockdown, BAPN treatment reduced proliferation of murine, but also human OS cell lines in vitro (Supplementary information, Fig. S7a). Cohorts of H2-*c-fos*LTR mice were next treated with BAPN in a therapeutic setting and OS development followed over time (Fig. 6a). Micro-CT analyses revealed a similar number of OS in the vehicle (PBS) and BAPN group (Supplementary information, Fig. S7b), but the average tumor size (Supplementary information, Fig. S7c) and tumor burden (Fig. 6b) were significantly reduced in BAPN-treated H2-*c-fos*LTR mice. Individual tumor analyses further indicated that tumors from BAPN-treated mice grew slower (Fig. 6c). Histological analyses at the end-point revealed that OS from BAPN-treated mice had less bony/osteoblastic and more fibroblastoid characteristics (Supplementary information, Fig. S7d) with reduced collagen packing (Fig. 6d, e), similar to what was observed when Wls was inactivated. Importantly, BAPN treatment also reduced tumor volume in mice bearing orthotopic xenografts of human OS cells (Supplementary information, Fig. S8a–e). Furthermore, while LOXL2 expression in the tumors was unchanged (Supplementary information, Fig. S9a), tumor collagen packing density (Supplementary information, Fig. S9a, b) and lung colonization (Supplementary information, Fig. S9c) were decreased in BAPN-treated LM7-derived OS-bearing mice. In addition, tumor-induced bone destruction occurring in 143b orthotopic xenografts was diminished in BAPN-treated mice (Supplementary information, Fig. S9d). Finally, H2-*c-fos*LTR mice were treated with a specific Loxl2 blocking antibody AB0023 (αLoxl2)^58^ in a therapeutic setting (Fig. 6f). Micro-CT analyses revealed significantly fewer (Fig. 6g) and smaller (Fig. 6h; Supplementary information, Fig. S9e) OS in αLoxl2-treated mice compared to the group treated with IgG. These data indicate that targeting Loxl2, but possibly also lysyl oxidases, is a therapeutic option in OS to enhance the efficacy of the current chemo-therapies. In support of this notion, combining BAPN with cisplatin had a stronger anti-proliferative effect on murine or human OS cell lines than either treatment alone (Supplementary information, Fig. S9f).

### c-FOS, WNT7B/9A and LOXL2 are co-expressed in human OS samples

We next assessed the correlation between WNT and LOXL2 mRNA expression using publicly available datasets for human OS. A cohort of 98 OS patients analyzed by RNA-seq was analyzed for WNT7B and WNT9A expression in the tumor and four groups were generated according to the expression of the two genes around their respective means (Supplementary information, Fig. S10a). LOXL2 mRNA expression was next computed for each group. As shown in Supplementary information, Fig. S10b, the highest LOXL2 mRNA expression was observed in the group expressing high WNT7B and high WNT9A (H/H). Consistent with the more robust induction of Loxl2 by Wnt7b observed in mouse cells, LOXL2 was also significantly increased in the WNT7B high (L/H) group.

We next assessed FOS, WNT7B, WNT9A and LOXL2 expression in human OS by (IHC using tissue microarrays (TMAs) from two independent sources: commercially available (US Biomax) and a TMA from the University Clinic of Navarra TMA (UCN). We also included an analysis of WNT7B and WNT9A mRNA by in situ hybridization (ISH) in the US Biomax TMA. Serial sections were stained with each antibody or ISH probe and positivity was scored in a semi-quantitative, blinded manner. Consistent with the data mining results and despite the smaller sample size, LOXL2 protein (IHC) expression showed a good correlation with WNT7B, and to a lesser extent WNT9A, mRNA (ISH) expression (Supplementary information, Fig. S10c, d).

**Fig. 7:**
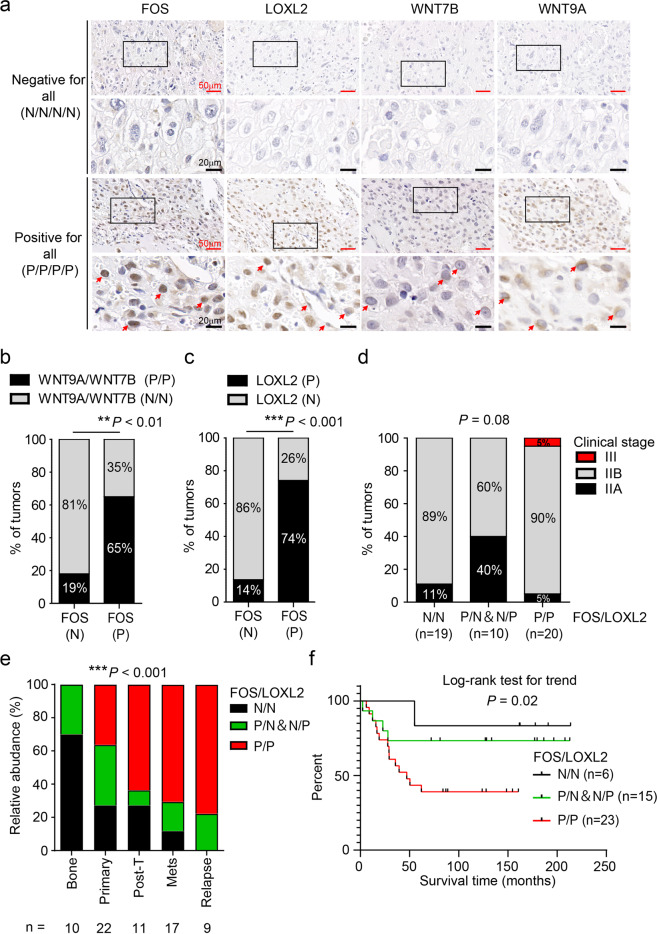
LOXL2 is co-expressed with WNT7A/WNT9A and FOS in human OS and FOS/LOXL2 double positivity is predictive of OS patient survival. Two independent tissue microarrays (TMA)/patient cohorts were employed. US Biomax TMA (OS802c) (**a**–**d**), University Clinic of Navarra TMA (UCN) (**e**, **f**). **a** Representative IHC images of FOS/LOXL2/WNT9A/WNT7B-quadruple negative (N/N/N/N) and -quadruple positive (P/P/P/P) in human OS TMA. Positive cells are indicated by red arrows. **b** Quantification of FOS-positive and -negative OS that are either WNT7B/WNT9A double-positive or -negative in a human OS TMA (Biomax, *n* = 49). **c** Quantification of FOS-positive and negative-OS that are either LOXL2-positive or -negative in a human OS TMA (Biomax, *n* = 49). **d** Correlation of FOS and LOXL2 IHC groups with OS clinical stage. For **b**–**d**, **P < 0.01, ***P < 0.001 and *P* = 0.08 by Fisher’s exact test. Brackets indicate the number of patients. **e** Relative abundance of FOS/LOXL2 positivity in OS analyzed by IHC in the UCN cohort according to patient diagnosis (primary tumor, post-treatment, metastatic or recurrent osteosarcoma). Bone, normal bone; Primary, primary tumor; Post-T, post-treatment; Mets, metastases; Relapse, relapsed tumor. *P* value is determined by χ^2^ statistics. **f** Overall survival of OS patients according to FOS/LOXL2 positivity (all patients included). Data are analyzed by Log-rank test and patient number is in brackets. P positive, N negative, N/N double-negative, P/N and N/P single positive, P/P double-positive.

Protein (IHC) analyses of 49 OS samples in the US Biomax TMA (Fig. 7a) indicated that 27 (55%) displayed nuclear FOS immunoreactivity, which is consistent with previous reports,^55^ while 23 (47%) were positive for LOXL2 in the nucleus, cytoplasm and ECM (Table 1). Importantly, the majority of FOS-positive samples were also WNT7B/WNT9A double-positive (Fig. 7b) by IHC. There was also a very good correlation between c-FOS and LOXL2 expression (Fig. 7c and Table 1) as the majority (39/49) of OS samples were either double-positive for c-FOS and LOXL2 or double-negative for the two proteins, whereas only few samples appeared single-positive and expressed either only c-FOS (7/49) or only LOXL2 (3/49). Furthermore, FOS and LOXL2 double-positivity also correlated with worse clinical stage (Fig. 7d). FOS and EGFR expression correlated in OS samples (Supplementary information, Fig. S10e), as previously reported,^27^ and LOXL2-immunoreactivity was over-represented in the EGFR/FOS double-positive samples compared to double-negative OS (Supplementary information, Fig. S10f). Of note, none of the abovementioned correlations between EGFR, FOS, WNT7B/WNT9A and LOXL2 expression was observed, when examining 27 chondrosarcoma (CS) patient samples that were included in the US Biomax TMA (Supplementary information, Fig. S11, Table 1 and data not shown), indicating that our findings are likely specific to OS.

**Table 1:** Summary of LOXL2 and FOS immunostaining of human OS.

Panel a	Osteosarcoma (*n* = 49)					Chondrosarcoma (*n* = 27)				
	FOS positive		FOS negative			FOS positive		FOS negative		
	Cases	% cases	Cases	% cases	*P* value	Cases	% cases	Cases	% cases	*P* value
Total	27/49	55%	22/49	45%		15/26	58%	11/26	42%	
LOXL2 positive	20/27	74%	3/22	14%	0.00002	14/15	93%	8/11	73%	0.165
LOXL2 negative	7/27	26%	19/22	86%		1/15	7%	3/11	27%	

Panel b	LOXL2 positive		LOXL2 negative			LOXL2 positive		LOXL2 negative		
	Cases	% cases	Cases	% cases	*P* value	Cases	% cases	Cases	% cases	*P* value
Total	23/49	47%	26/49	53%		24/27	89%	3/27	11%	
FOS positive	20/23	87%	7/26	27%	0.00002	14/24	58%	1/3	33%	0.165
FOS negative	3/23	13%	19/26	73%		9/24	38%	2/3	67%	
Localization of Loxl2										
Nuclear	3/23	13%				10/24	42%			
Cytoplasmic + ECM	8/23	34%				0/24	0%			
Nuclear + Cytoplasmic + ECM	12/23	52%				14/24	58%			

The table includes 49 Osteosarcoma (OS) and 27 Chondrosarcoma (CS) from the US Biomax TMA (OS802c). Semi-quantitative analysis of LOXL2 and FOS expression in OS and CS with the percentage of samples with positive or negative staining in each subgroup (see also Fig. 7a–d). Panel a: samples are divided into FOS-positive and -negative groups and further subdivided into LOXL2-positive and -negative subgroups. Panel b: samples are divided into LOXL2-positive and -negative groups and further subdivided into FOS-positive and -negative subgroups. The localization of the LOXL2 IHC signal is indicated. ECM extracellular matrix. *P* value is calculated using Fisher’s exact test.

IHC analyses using human OS tissues from the UCN cohort (48 OS patients, 153 tissue spots) were consistent with the data obtained using the Biomax TMA. WNT7B/WNT9A double-positive samples were mostly found in the FOS-positive population (Supplementary information, Fig. S12a), two thirds of Fos-positive samples were also LOXL2-positive (Supplementary information, Fig. S12b) and LOXL2 positivity was more frequently observed in samples positive for FOS plus one or two WNT proteins (Supplementary information, Fig. S12c). Importantly, the proportion of FOS/LOXL2 double-positive cases steadily increased within the OS samples along diagnosis: from 35% of FOS/LOXL2 double-positive primary tumors to almost 80% FOS/LOXL2 double-positive relapsed OS (Fig. 7e). FOS/LOXL2/WNT positivity correlated with worse prognosis. Patients positive for FOS, LOXL2, WNT7B and/or WNT9A exhibited shorter overall survival compared to the rest of patients (Supplementary information, Fig. S12d). Remarkably, FOS and LOXL2 double positivity was sufficiently predictive of patient survival (Fig. 7f; Supplementary information, Fig. S12e, f). These data indicate that FOS, WNT7B/WNT9A and LOXL2 are co-expressed in a substantial fraction of human OS, further supporting the diagnostic, prognostic and/or therapeutic relevance of our findings.

## Discussion

OS is the most common type of bone cancer with heterogeneous histological and molecular profiles. Despite a reasonable success rate combining surgery and chemotherapy currently as the standard of care, OS has one of the lowest pediatric cancers survival rates. It is therefore important to understand how the disease develops and the functional significance of its heterogeneity to identify better prognostic markers and novel therapies aimed to improve the efficacy of the current treatment strategies, especially for unresectable, recurrent and metastatic OS.

OS cells modify their microenvironment by secreting several ECM components, including collagens and collagen-modifying enzymes. Patients with fibroblastic OS respond better to therapy than those with chondroblastic or osteoblastic histopathology.^10–12^ It is therefore plausible that molecular determinants controlling the histological characteristics of OS as well as cell-ECM and cell-cell interactions in the tumor microenvironment are important contributors to OS biology, possibly involved in therapeutic response.

In the present study, we observed reduced incidence and progression of OS associated with increased fibroblastic characteristics when Wnt signaling was genetically reduced in osteoblast/OS cells in the H2-*c-fos*LTR genetic mouse model for OS, suggesting that autocrine or paracrine Wnt signaling is an important contributor to OS pathology. Herein we identified *Wnt9a* and *Wnt7b*, two Wnt ligands involved in a context-dependent canonical and non-canonical Wnt signaling,^53,59,60^ as important modulators of OS development in mouse models, human xenografts and patient samples.

While mutations of Wnt target genes and/or Wnt signaling-related genes have been associated with OS, the mechanisms by which Wnt signaling operates in OS has been a matter of debate. Some studies suggest that Wnt/β-catenin signaling promotes OS growth, whereas others come to contrasting conclusions.^43,61–64^ The function of non-canonical Wnt pathways and ligands in OS is even less studied, despite the well-established importance of non-canonical Wnt pathways/ligands in normal bone development and homeostasis.^65^ Only one study noted increased mRNA of *WNT7b* in human OS cell lines,^61^ while another reported that the prototypical non-canonical Wnt ligand Wnt5a promotes the migration and invasion of a human OS cell line in vitro.^66^

**Fig. 8:**
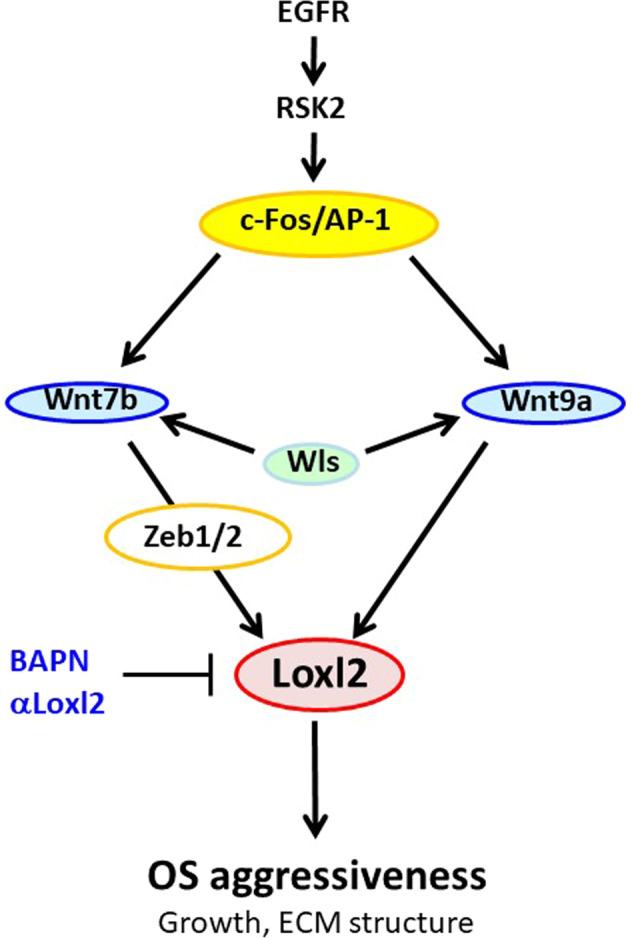
Schematic representation of the Fos/AP-1-Wnt7b/Wnt9a-Loxl2 axis operating in OS formation. Wnt9a and Wnt7b are two novel c-Fos/AP-1 target genes operating in an autocrine, and possibly paracrine, fashion in mouse and human OS to induce the expression of the collagen cross-linking enzyme Loxl2, thereby modulating collagen packing and impacting on osteoblast differentiation and tumor aggressiveness. c-Fos protein expression and stability is maintained through EGFR and its downstream kinase RSK2 and the new regulatory pathway can be therapeutically targeted by inhibiting Wnt secretion (Wls) and/or Loxl2 activity (BAPN or blocking antibodies), to benefit OS patients.

Screening regulators and downstream effectors, we demonstrate that *Wnt9a* and *Wnt7b* are two novel c-Fos/AP-1 target genes operating in an autocrine, and possibly paracrine, fashion to induce Loxl2 expression, thereby modulating collagen packing and impacting on osteoblast differentiation and tumor pathology. c-Jun/AP-1 was previously shown to modulate murine *Wnt9a* expression in the developing joint primordium through binding to a different *Wnt9a* promoter region.^67^ On the other hand, c-Fos protein expression and stability is maintained through EGFR signaling and its downstream kinase RSK2 and both are required for c-Fos-dependent OS development^26,27^ with c-Jun likely being the essential AP-1 dimerizing partner^68^ (Fig. 8). However, direct transcriptional targets of c-Fos modulating OS formation or histopathology have not been validated. Our “target validation” suggest that therapeutic strategies targeting Wls or Wnt9a/Wnt7b systemically to modify OS histology toward a favorable outcome might be applicable in the clinic. Such strategies could include WNT974, a small compound inhibiting the Wnt acyltransferase Porcupine, currently in phase I/II clinical trials for colorectal, squamous cell and head and neck cancers (NCT02278133 and NCT02649530). Since Porcupine-mediated Wnt acylation is essential for Wnt binding to Wls, and therefore Wls-mediated Wnt secretion, these clinical trials should also be informative on the possible adverse effects of systemically inhibiting Wnt secretion.

The lysyl oxidase family of enzymes (encoded by *LOX* and *LOXL1*-*4*) catalyze the final reaction required for collagen and elastin cross-linking, an essential step to ensure the structural integrity and function of several tissues including bone.^69^ Loxl2 has been correlated with poor prognosis of several solid cancers,^58,70,71^ but despite the important contribution of collagen to the structure and material properties of bony tissues, Loxl2 was never examined in the context of OS. Our data strongly support a positive correlation between FOS, WNT7B/WNT9A and LOXL2 and provide multiple lines of evidence that LOXL2 expression in FOS-positive OS cells is a functionally relevant determinant of OS in experimental models and human tissues. Interestingly, an opposite function has been proposed for the prototypic member of the family; Lox promoter polymorphisms were associated with OS risk,^72^ Lox expression was decreased in OS samples and Lox had tumor suppressor activity in OS cell lines.^73^ Nevertheless, our in vivo experiments using H2-*c-fos*LTR mice and human and mouse OS xenografts indicate that tumor size was significantly reduced and collagen packing density substantially affected when Loxl2 was inhibited either pharmacologically together with Lox or specifically by shRNA or by blocking antibodies. Therefore, Loxl2 is likely a tumor promoter in OS. Since ECM stiffness affects osteoblast differentiation, for example through the transcription factor YAP/TAZ,^74^ the collagen cross-linking activity of Loxl2 could favor bone formation in the tumor and promote the more aggressive osteoblastic phenotype. Furthermore, we observed in orthotopic transplantation models that lung colonization and bone destruction by tumor cells was diminished by targeting Lox/Loxl2, indicating that Loxl2 expression by OS cells might also be important for these deleterious aspects of OS, whether these are mediated by collagen cross-linking or by the recently described non-enzymatic functions of Loxl2.^69,75^ This result predicts that Loxl2-inhibiting agents, a number of which have been already tested in clinical trials for other diseases, will enhance the therapeutic efficacy of current OS standard of care, especially for unresectable, metastatic and/or recurrent OS. In addition, FOS/LOXL2-double positivity in a primary tumor could help identify those early stage patients, who might also benefit from anti-Loxl2 or anti-Wls targeted therapies together with chemotherapy.

In summary, these results describe a new regulatory pathway whereby c-Fos controls Wnt7b and Wnt9a transcription in OS and consequently Wnt7b/9a-induced expression of the collagen cross-linking enzyme Loxl2 (Fig. 8). This promotes structural alterations of the tumor/matrix as well as fibroblastic features of mouse and human OS. These findings uncover new therapeutic strategies for targeting Wnt and/or Loxl2 with the prospect to benefit OS patients.

## Materials and methods

A complete list of plasmids, primers and antibodies used in this study is provided in Supplementary information, Tables S1 and S2.

### Mice

The H2-*c-fos*LTR,^25^ *Wls floxed*^49^ and Osx-tTA-gfp::cre^50^ mice and alleles were previously described. NSG mice (NOD-SCID IL-2Rγ^−/−^, The Jackson Laboratory, Stock No: 005557) were purchased from the CNIO or the MUW animal facility. H2-*c-fos*LTR mice were maintained on pure C57BL/6J background while *Wls*^flox/flox^, Wls^WT^-OS and Wls^ΔOB^-OS mice were maintained on mixed (C57BL/6×129S6/Sv) background. As no gender-related difference was observed in the analyses of the H2-*c-fos*LTR mice and derivatives, data from females and males were combined, while only male NSG were used for xenografts. Randomized block design was used to organize the experimental cohorts. Mice were housed in Specific Pathogen-Free environment with free access to food and drink. All animal experiments were conducted according to institutional policies and national and European guidelines.

### BAPN and anti-Loxl2 antibody treatment in H2-*c-fos*LTR mice

Five-week-old mice were randomized and injected intraperitoneally during 15 weeks, either three times a week with BAPN (250 mg/kg body weight) or PBS or twice a week with IgG or anti-Loxl2 antibody AB0023 (Gilead Sciences SA, 30 mg/kg body weight).

### Intra-tibial orthotopic tumor cell injection, in vivo Loxl2 knock-down and BAPN treatment

Ten-week-old NSG males were anesthetized and H2-*c-fos*LTR or human OS cell lines injected (10^6^ cells in 25 μL PBS) through the knee to the bone marrow cavity of the left proximal tibia using an insulin syringe. Three days later, mice were randomized and injected intra-peritoneally three times a week with IPTG (0.55 μM) or vehicle (PBS) during 18 days. BAPN (250 mg/kg body weight) was injected intraperitoneally three times a week for 15 days starting 5 days after OS cell injection. The volume of tumor-bearing (affected) and contralateral (unaffected) legs was quantified at end point by Micro-CT. Differences in tumor burden were independently confirmed by weighing the dissected legs and subtracting the weight of the unaffected leg from that of the tumor-bearing leg for each mouse.

### Longitudinal and end point Micro-CT

Micro-CT was performed either on mice anesthetized with a continuous flow of 1%–3% isoflurane/oxygen mixture (2 L/min) or (at end point) on isolated and hindlimbs and pelvic region. Longitudinal tumor growth was measured every 5 weeks on live H2-*c-fos*LTR mice and the same tumors followed over time. OS affected femur, tibia, pelvis and spine and none of the conditions assessed affected tumor localization. A GE eXplore Locus Micro-CT scanner (GE Healthcare, London, Canada) with an isotropic resolution of this instrument of 45 μm was used. The Micro-CT image acquisition consisted of 400 projections collected in one full rotation of the gantry in ~10 min. Image acquisition was not respiratory-gated. The X-ray tube settings were 80 kV and 450 μA. The resulting raw data were reconstructed to a final image volume of 875 × 875 × 465 slices at (93 μm)^3^ voxel dimensions. Two-dimensional (2D) x-ray images of the tumor/bone were extracted from the data and followed by a digital three-dimensional (3D) reconstruction. Tumor volume (mm^3^) and tumor bone mineral density (BMD, mg/cc) were quantified from the reconstructions using the dual-licensed 3D image viewer GE MicroView software v2.2 with Advanced Bone analysis and CT images from OS-free WT/control littermates were used to compute the parameters of a normal bone structure. Tissue density of the tibia/femur trabecular bone region of OS-free mice was used as a cut-off value to distinguish between bone/OS and other tissues, such as muscle, connective tissues, fat and bone marrow in an unbiased manner. Abnormally growing regions in the bones of H2-*c-fos*LTR mice were always mineralized, even if sometimes in a heterogeneous fashion. Only CT-visible H2-*c-fos*LTR OS greater than 0.1 mm^3^ were included in the analysis and macroscopical observation and histology at end-point confirmed that OS were correctly identified. For human and mouse xenografts with little, unmineralized tumors, tumor volume was estimated by measuring the volume of the whole injected bottom hindleg and substracting the volume of a similarly measured contralateral non-injected leg. To generate two colors 3D reconstruction images of human and mouse OS xenografts acquisition files were exported from GE MicroView in DICOM format to the open source free software platform for medical image informatics 3D Slicer. 3D reconstruction of the tumor-bearing and contralateral leg was carried out in 3D Slicer by segmenting the images using greyscale thresholds corresponding to the tissue density (tumors and muscles) and allocating a different color to each image segment according to tissue type.

### Immunohistochemistry

After Micro-CT, fixed hindlimbs and pelvis were dissected, decalcified with 18% EDTA (pH 8.0) during 2 weeks before paraffin embedding for hematoxylin/eosin staining and/or IHC. Immunohistochemistry/immunofluorescence was performed on 5 μm thick sections. Slides were deparaffinized using xylene or citrus reagents and subsequently bathed in decreasing alcohol concentrations (100%, 96%, 70%, ethanol) followed by water washes. Deparaffinized tissue sections were treated with H_2_O_2_ for 30 min and antigen retrieval was carried out using citrate buffer and a pressure cooker for 20 min. After permeabilization by 0.1% Triton X-100/PBS for 10 min, non-specific binding was blocked with 10% normal serum/PBS. Tissue sections were incubated with primary antibodies (Supplementary information, Table S1) overnight at 4 °C. Biotin/streptavidin amplification and HRP-based chromogen detection (VECTASTAIN ABC Kit) was used for IHC following the manufacturer’s instructions, while for immunofluorescence sections were incubated with Alexa Fluor® 594 goat anti-rabbit IgG (H + L) for 1 h.

### In situ hybridization

Non-radioactive in situ hybridizations were performed using digoxigenin-labeled antisense ribo-probes for human WNT7b and WNT9a generated against nucleotides 1189–1825 and 1779–2407 based on the NCBI reference sequences NM_058238 and NM_003395, respectively. Hybridizations were performed as previously described, with the modification that the hybridization temperature was lowered to 55 °C.^76^

### Picrosirius red staining

Deparaffinized tissue sections were stained with Direct Red 80 in Picrosirius acid (Sigma) for 1 h at room temperature (RT). After two washes in 0.55% acetic acid in distilled water, samples were dehydrated in absolute alcohol, incubated in xylene and mounted using permanent non-aqueous mounting media (Entellan^®^ new, Sigma-Aldrich). Whole slides were acquired with a slide scanner (AxioScan Z1, Zeiss) using both bright-field and polarized light. Regions of interests were selected in the polarized light images using the analysis module included in the image acquisition software. A dedicated script was created for collagen quantification where positivity was quantified in three phases (phase 1, red area; phase 2, yellow area; phase 3, green area) and compared with the total area (total area = phase 1 + phase 2 + phase 3).

### EdU assay

Fifteen-week-old mice were injected intraperitoneally with 100 µL of a 10 mg/mL EdU solution dissolved in sterile PBS. Mice were sacrificed 4 h after injection and tumors/bones were dissected, fixed in 10% formalin, decalcified with 18% EDTA (pH 8.0) for 2 weeks and embedded in paraffin. EdU-labeled cells were detected on deparaffinized sections using a Click-iT^®^ Alexa Fluor 647 Imaging Kit (Thermo Fisher) according to the manufacturer’s protocol and quantified using the ImageJ plugin for cell count or ImmunoRatio (ImageJ bundled with 64-bit Java 1.8.0_112).

### Cell culture

The OS cell lines derived from H2-*c-fos*LTR mice were previously described^25^ and primary mouse OS cells were isolated and cultured from H2-*c-fos*LTR, Wls^WT^-OS and Wls^ΔOB^-OS mice following a similar protocol. The human OS cell lines 143b and LM7^77^ were purchased from ATCC and provided by Dr. Eugenie S. Kleinerman (M.D. Anderson Cancer Center), respectively. L cells, Wnt3a-expressing L cells and pCAGG Wnt9a-HA were provided/generated in the Hartmann lab, pLNC-Wnt7b-HA, pLNC-Wnt3a-HA and pLNC-Wnt5a-HA (Addgene #18037, #18030 and #18032) by Dr. Jan Kitajewski^78^ and pcDNA-WNT7B and pcDNA-WNT9A (Addgene #35915 and #35918) by Dr. Marian Waterman.^79^ Human^80^ and murine^81^ c-Fos expression plasmids were previously described (see also Supplementary information, Table S2). MC3T3-E1 cells, 293T cells, L cells and murine and human OS cells were cultured in DMEM supplemented with 10% FCS (Sigma). MC3T3-E1, OS cell lines and L cells were transfected with the different Wnt or Fos expression constructs using FuGENE HD^®^ transfection reagent (Invitrogen) and the cells subsequently processed either at 48 h after transfection of plasmid (MC3T3-E1 and OS cell lines) or after 14 days of selection with 200-800 μg/mL G418, as a source of conditioned medium (CM). Established L cells expressing Wnt3a, Wnt7b-HA and Wnt9a-HA were next cultured for 2–3 days without selection, CM was collected and replaced with fresh medium that was harvested 2–3 days later. The two batches of CM were mixed 1:1 and filtered through 0.45 μm filters before use.

### shRNA-mediated gene silencing

Tet-pLKO-neo was a gift from Dr. Dmitri Wiederschain^82^ (Addgene #21916). The shRNA expression vectors pLKO*-*puro*-*IPTG-3xLacO (Sigma) and Tet-pLKO*-*neo were digested with *Age*I and *Eco*RI and, following the provider's instructions, purified and ligated with two annealed synthetic oligonucleotides selected from the Broad Institute Gene Perturbation Portal web resource (https://portals.broadinstitute.org/gpp/public/): Murine c-Fos: TRCN0000042679, murine Loxl2: TRCN0000076711 and human FOS: TRCN0000016007. The resulting lentiviral shRNA expression vectors were co-transfected with pVSV-G and pCMV-Δ89 vectors into 293T cells; culture supernatants collected and used to infect OS cell lines. Cells were selected with 2 mg/mL puromycin (C3 cells) or with 500-800 μg/mL G418 (143b and LM7 cells) for 7 days and used for subsequent experiments. The generation and characterization of 143b-*FOS* shRNA cells were previously described.^27^

### Luciferase reporter assay

Murine Wnt9a and Wnt7b luciferase reporters were constructed by cloning a PCR-amplified fragment of mouse genomic DNA, containing the proximal and distal TRE-like elements, into the pGL4.23 luciferase reporter (Promega, E8411). The cloned fragments are depicted in Supplementary information, Fig. S3c: −520 to −1445 and from −925 to −218 base pairs relative to transcription start site, respectively. The Fra1 reporter was previously described.^83^ MC3T3-E1 cells were plated at 5 × 10^4^ cells/well in 24-well dishes in triplicate and co-transfected with 0.1–0.5 μg CMV-c-Fos,^81^ 0.5 μg of the luciferase reporter and 0.025 μg of phRG-Renilla internal control (Promega) using FuGENE HD^®^ (Roche). Luciferase activity was quantified using the Dual-Luciferase Kit (Promega) with a 1420 Multilabel Counter Victor 3 (Perkin Elmer).

### Colony formation assay

Twenty-five cells/well were seeded in six-well plates and cultured with DMEM containing 10% FBS with/without IPTG (30–120 nM) for 2 weeks at 37 °C. Cells were fixed with 4% paraformaldehyde, washed with PBS and stained with 0.05% crystal violet for 30 min and washed with water.

### MTT assay and XTT assay

Two thousand and five hundred cells/well were seeded in 96-well plates and cultured for 96 with or without BAPN (10^−6^–10 mM). 25 μl of MTT reagent (5 mg/mL) was next added to each well and incubated for 4 h at 37 °C. Wells were washed with PBS and the remaining crystals were dissolved in dimethyl sulfoxide. Absorbance was evaluated at 570 nm (background 630 nm). The assays were performed in triplicate. For XTT assay (Merck), 5000 cells/well were seeded in 96-well plates, cultured for 48 h with 5 mM BAPN, 100 μM cisplatin and both compounds and processed according to the manufacturer’s instructions.

### RNA isolation and qPCR

Total RNA was isolated using TRI Reagent (Sigma-Aldrich), complementary DNA was synthesized using Ready-To-Go-You-Prime-First-Strand Beads (GE Healthcare) or GoScript™ Reverse Transcription Mix, Oligo(dT) (Promega) and qPCR used GoTaq qPCR Master Mix (Promega) and Eppendorf fluorescence thermocyclers, all according to the manufacturer’s instructions. The 2^ΔΔCT^ method was used to quantify the amplified fragments. Expression levels were normalized using at least one housekeeping gene. Primer sequences are listed in Supplementary information, Table S1.

### Immunoblotting

Cells were lysed in radio-immunoprecipitation assay (RIPA) buffer (25 mM Tris-HCl/150 mM NaCl/1% NP-40/0.1% SDS/0.5% sodium deoxycholate/1 mM EDTA, pH 7.5) supplemented with complete™, EDTA-free Protease Inhibitor Cocktail (Roche) and PhosSTOP™ (Roche) and centrifuged at 13,000× *g* for 10 min at 4 °C. Cell lysates were subjected to SDS-PAGE on 5%–20% acrylamide gels and proteins were transferred to nitrocellulose membranes. Membranes were blocked with 5% low-fat milk; 1% Tween-20 in PBS and incubated with primary antibodies overnight at 4 °C (Supplementary information, Table S2) and with the appropriate HRP-conjugated secondary antibodies. Enhanced chemiluminescence (WBLUR0100 Immobilon Crescendo Western HRP substrate, Merck) and by X-ray film (Kodak) was used for visualization.

### ChIP

To analyze c-Fos binding to the promoter region of Wnt7b and Wnt9a, two 15 cm dishes of C3-shRNA c-Fos and C3-shRNA non-target cell lines were cultured with fresh DMEM containing 500μM IPTG (Cliniscience) for 3 days. PBS was used as vehicle control. Cells were fixed by adding formaldehyde directly to the culture dish to 1% final concentration, incubated for 10 min at 37 °C and the cross-linking reaction was quenched by adding glycine to a final concentration of 0.125 M. Cells were washed with ice-cold PBS and scraped off the culture dishes and harvested by mild centrifugation. Cell pellets were resuspended in 2 mL lysis buffer 1 (50 mM HEPES-KOH, pH 7.5, 140 mM NaCl, 1 mM EDTA, 10% glycerol, 0.5% NP-40, 0.25% Tritone X-100, protease inhibitors) and incubated at 4 °C on a rotator for 10 min. After centrifugation, cell pellets were resuspended in 2 mL of lysis buffer 2 (10 mM Tris-HCl, pH 8.0, 200 mM NaCl, 1 mM EDTA, 0.5 mM EGTA, protease inhibitors) and incubated at 4 °C on a rotator for 10 min. After centrifugation, cell pellets were resuspended in 1.9 mL of lysis buffer 3 (10 mM Tris-HCl, pH 8.0, 100 mM NaCl, 1 mM EDTA, 0.5 mM EGTA, 0.1% Sodium-Deoxycholate, 0.5% N-lauroylsarcosine, proteinase inhibitors) and 200 μL of 10% Triton X-100 (1/10 volume) was added and then sonicated using a Covaris-S2 Focused Ultrasonicator (Covaris). After centrifugation, the cell lysate was pre-cleared by with Dynabeads^TM^ Protein G (Thermo Fisher) and 200 μL (1/10 volume) were set apart as input DNA. c-Fos antibody (9F6, Cell Signaling Technology) was added to the remaining cell lysates and incubated for 16 h at 4 °C with rotating. Dynabeads^TM^ Protein G was added and incubated at RT for 1 h. Beads-antibody complexes were collected, washed with low-salt wash buffer, high-salt wash buffer, LiCl buffer and TE buffer. Protein-DNA complexes were eluted in elution buffer (50 mM Tris-HCl, pH 8.0, 10 mM EDTA, 1% SDS). The eluate and the input aliquot was reverse cross-linked at 65 °C for 6 h and sequentially treated with 2 µL of RNaseA at 37 °C for 2 h and 2.5 µL of Proteinase K at 55 °C for 2 h. The 400 μL of Phenol/Chloroform/Isoamyl alcohol was added to the eluate, the input was centrifuged at maximal speed at RT for 10 min and the aqueous layer was transferred to a new tube. 16 µL of 5 M NaCl, 1.5 μL of 20 µg/µL glycogen and 800 µL of 100% EtOH were added to the eluate and incubated at −80 °C for 2 h and then centrifuged at 4 °C for 10 min. The pellets were washed with 500 μL of 80% EtOH, centrifuged at 4 °C for 5 min and left to dry. Finally, DNA pellets were resuspended in 200 uL of TE buffer and incubated at 65 °C for 15 min and 1–2 µL was used for qPCR. Primers used for ChIP qPCR are listed in Supplementary information, Table S2.

### Human mRNA expression data

Publicly available TARGE-OS RNA-seq datasets were downloaded from TARGET Data Matrix (https://ocg.cancer.gov/programs/target/data-matrix) (Office Cancer Genomics, National Cancer Institute). Transcripts Per Kilobase Million (TPM) for WNT7B, WNT9A and LOXL2 were extracted and log2(TPM + 1) is plotted as the expression level. The mean-log2(TPM + 1) for each gene was used to define the respective high or low groups and generate the four cohorts: L/L (low WNT7B and low WNT9A), H/H (high WNT7B and high WNT9A), H/L and L/H (with high WNT7B and low WNT9A and vice versa).

### Human OS TMA

Consecutive sections from a commercially available tissue microarray (TMA) (US Biomax Inc OS802c) and a TMA developed at the University Clinic of Navarra containing 233 tissue spots from 48 osteosarcoma patients^84^ were stained with antibodies against c-Fos (Santa Cruz Biochemistry, SC-52), Loxl2 (Abcam, ab96233), WNT7B (Sigma, SAB2701193) and WNT9A (Abcam, ab125957). Positivity in each stained core was graded blindly by two independent investigators and additionally with an automatized histology quantification program (Definiens Tissue Studio^®^ 4.0 or ZEN).

### Statistical analysis

Prism8 (GraphPad) was used for analyses and statistics. Statistical significance was determined using either paired or unpaired *t*-test (one- or two-tailed), or Mann-Witney test or Kruskal-Wallis test according to sample distribution, for comparing two or more groups of samples. One-way ANOVA or two-way ANOVA were performed for grouped or multivariate analysis, as appropriate. If the variances were significantly different, as measured by f-test, unpaired two-tailed Student’s *t*-test with Welch’s correction was applied. For analyses of more than two groups/time points (e.g. BAPN long-term treatment experiment), two-way ANOVA with Bonferroni post hoc test was applied. Differences in survival were plotted in Kaplan-Meier curve and were tested for statistical significance with log-rank (Mantel Cox correlation to compare two groups and test for trend to compare three groups). Unless otherwise specified, data are shown as mean ± SEM and a *P* value below 0.05 is considered statistically significant and indicated as *P < 0.05, **P < 0.01, ***P < 0.001.

## Supplementary information

Supplementary Figure S1

Supplementary Figure S2

Supplementary Figure S3

Supplementary Figure S4

Supplementary Figure S5

Supplementary Figure S6

Supplementary Figure S7

Supplementary Figure S8

Supplementary Figure S9

Supplementary Figure S10

Supplementary Figure S11

Supplementary Figure S12

Supplementary Table S1

Supplementary Table S2
