# Supplementary material for: Wnt signaling and Loxl2 promote aggressive osteosarcoma
Source: Cell Res. 2020 Jul 20;30(10):885–901. doi: 10.1038/s41422-020-0370-1 (PMC7608146; doi:10.1038/s41422-020-0370-1)
Supplement: Supplementary file 1 — Supplementary Figure S1 [file 41422_2020_370_MOESM1_ESM.pdf]

Supplementary information Figure S1

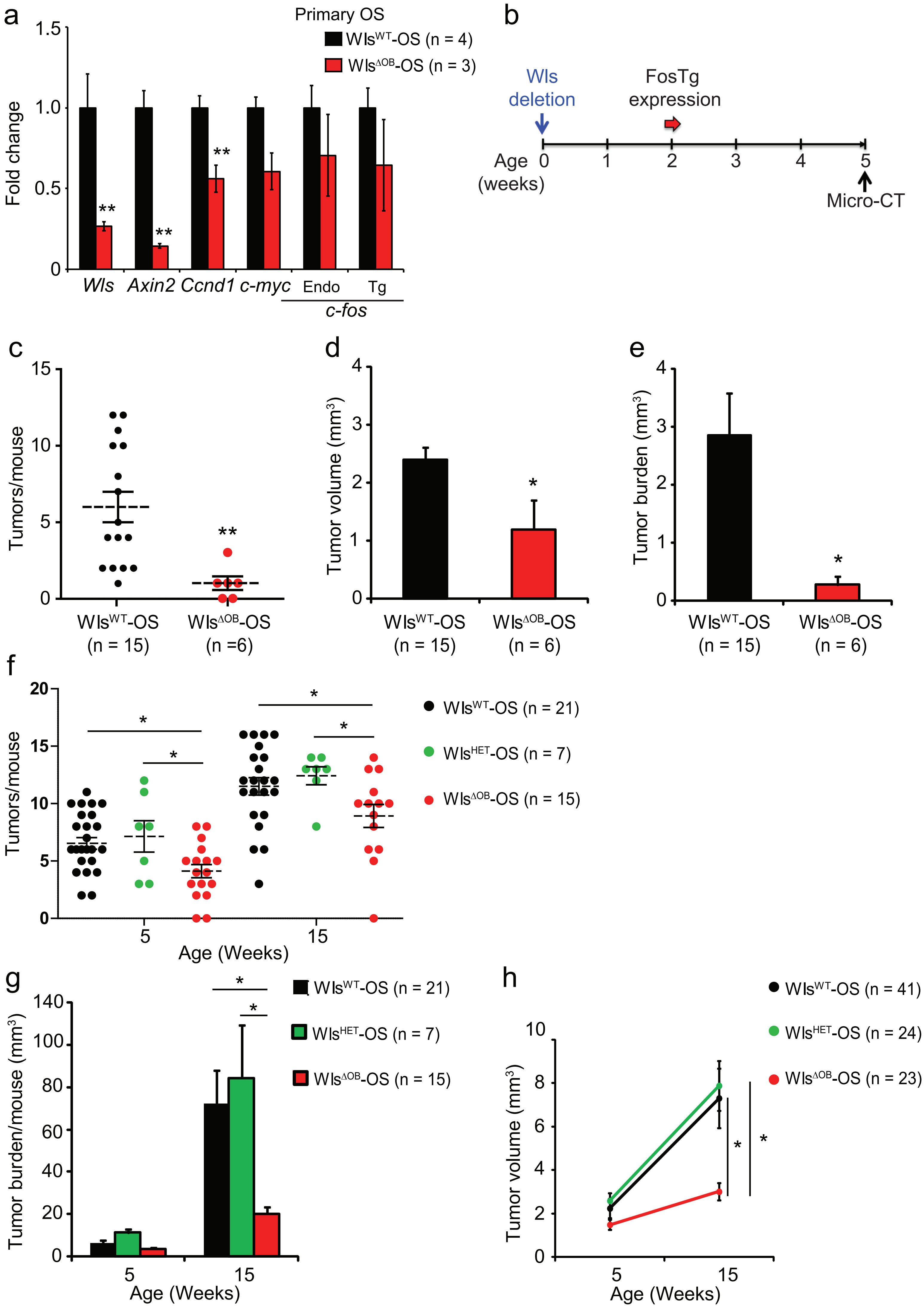

Supplementary information Figure S1. Wls deletion in osteoblast progenitors reduces c-Fos-induced OS

(a) qPCR analysis of *wls*, Wnt target genes and Fos mRNA species (endo= endogenous, Tg= transgene) in Wls^{WT}-OS and $Wls^{\Delta OB}$-OS primary OS cells **(b-e)** Tumor development in $Wls^{\Delta OB}$-OS when Dox is removed to inactivate *wls* at birth, before onset of FosTg expression **(b)**. Tumor number per mouse **(c)**, average tumor volume **(d)** and average tumor burden per mouse **(e)** by Micro-CT at 5 weeks in Wls^{WT}-OS mice (n = 15) and $Wls^{\Delta OB}$-OS (n = 6) mice. **(f-h)** Tumor development in Wls^{HET}-OS mice, with only one, inactivated *wls* allele compared to Wls^{WT}-OS and $Wls^{\Delta OB}$-OS: Dox was removed at 3 weeks of age and tumors were monitored at 5 and 15 weeks. Tumor number per mouse **(f)** and average tumor burden per mouse **(g)** in Wls^{WT}-OS (n = 21), $Wls^{HET/+}$-OS (n = 7) and $Wls^{\Delta OB}$-OS (n = 15) mice. **(h)** Individual tumor follow up over time of 41 Wls^{WT}-OS tumors, 24 Wls^{HET}-OS and 23 $Wls^{\Delta OB}$-OS tumors. Bar graphs and plots represent or include mean ± sem, respectively. *$P < 0.05$ and **$P < 0.01$.
