# Supplementary material for: Wnt signaling and Loxl2 promote aggressive osteosarcoma
Source: Cell Res. 2020 Jul 20;30(10):885–901. doi: 10.1038/s41422-020-0370-1 (PMC7608146; doi:10.1038/s41422-020-0370-1)
Supplement: Supplementary file 2 — Supplementary Figure S2 [file 41422_2020_370_MOESM2_ESM.pdf]

Supplementary information Figure S2

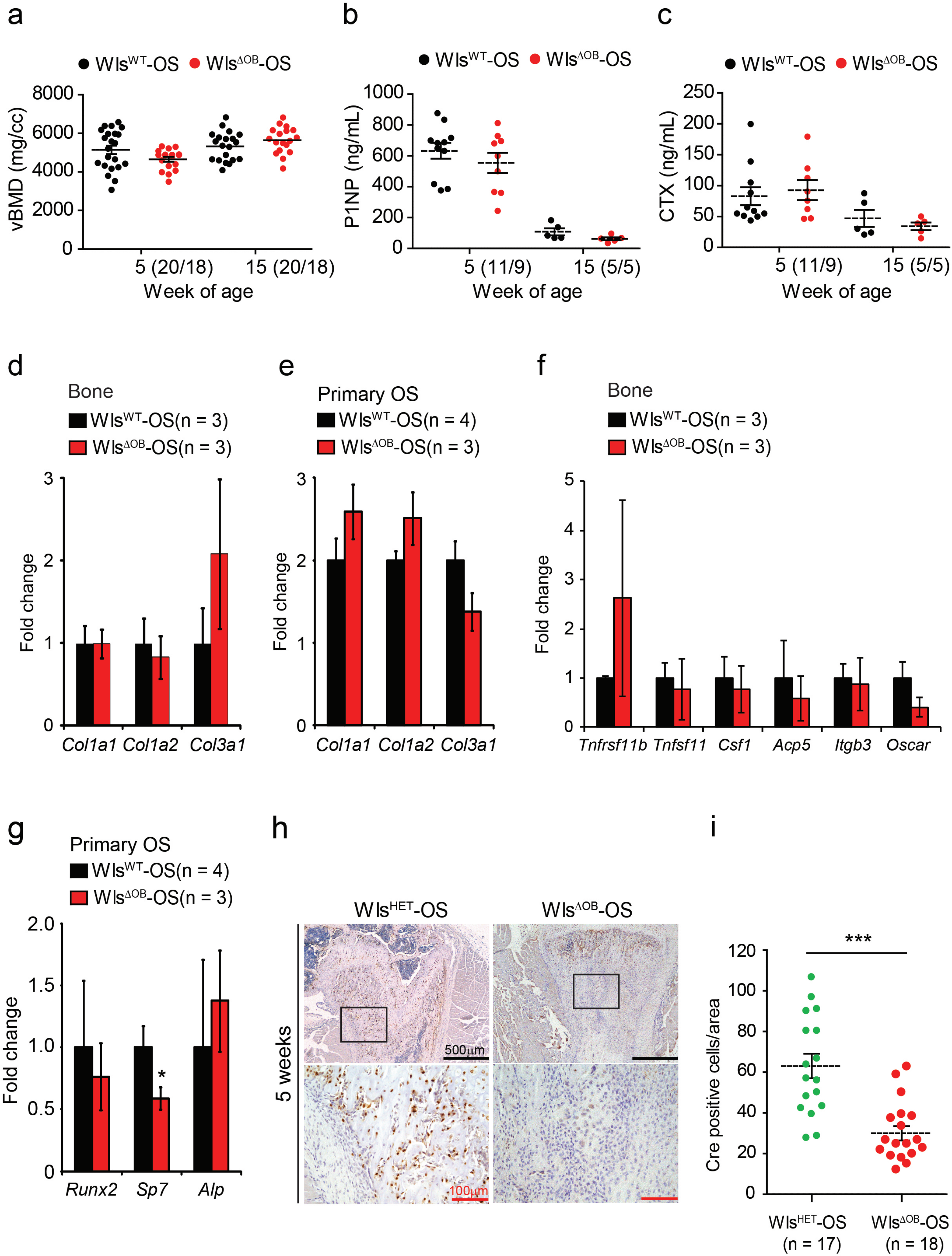

Supplementary information Figure S2. Wls deficiency in osteoblast progenitors affects OS pathology

(a) Micro-CT measurement of vBMD in Wls^{WT}-OS and $Wls^{\Delta OB}$-OS cortical bones at 5 and 15 weeks of age. The number of analyzed WT/ΔOB bones is indicated in parentheses beside each time point. **(b)** Serum P1NP and **(c)** CTX in Wls^{WT}-OS and $Wls^{\Delta OB}$-OS mice at 5 and 15 weeks of age. The number of analyzed mice is indicated in parentheses beside each time point. qPCR analysis of collagens in tumor-bearing bones from 5 week-old Wls^{WT}-OS and $Wls^{\Delta OB}$-OS mice **(d)** and in primary OS cells **(e)**. **(f)** qPCR analysis of osteoclastogenic and osteoclast marker genes in tumor-bearing bones from 5 week-old Wls^{WT}-OS and $Wls^{\Delta OB}$-OS mice. **(g)** qPCR analysis of osteoblast/osteocyte marker genes in primary OS cells. IHC analysis of Cre (as a reporter for Osterix promoter activity) in tumor-bearing sections from 15 weeks-old $Wls^{\Delta OB}$-OS and Wls^{HET}-OS mice. Representative images **(h)** and quantification **(i)** are shown. Bar graphs and plots represent or include mean ± sem, respectively. **P* < 0.05 and ****P* < 0.001.
