# Supplementary material for: Wnt signaling and Loxl2 promote aggressive osteosarcoma
Source: Cell Res. 2020 Jul 20;30(10):885–901. doi: 10.1038/s41422-020-0370-1 (PMC7608146; doi:10.1038/s41422-020-0370-1)
Supplement: Supplementary file 3 — Supplementary Figure S3 [file 41422_2020_370_MOESM3_ESM.pdf]

Supplementary information Figure S3

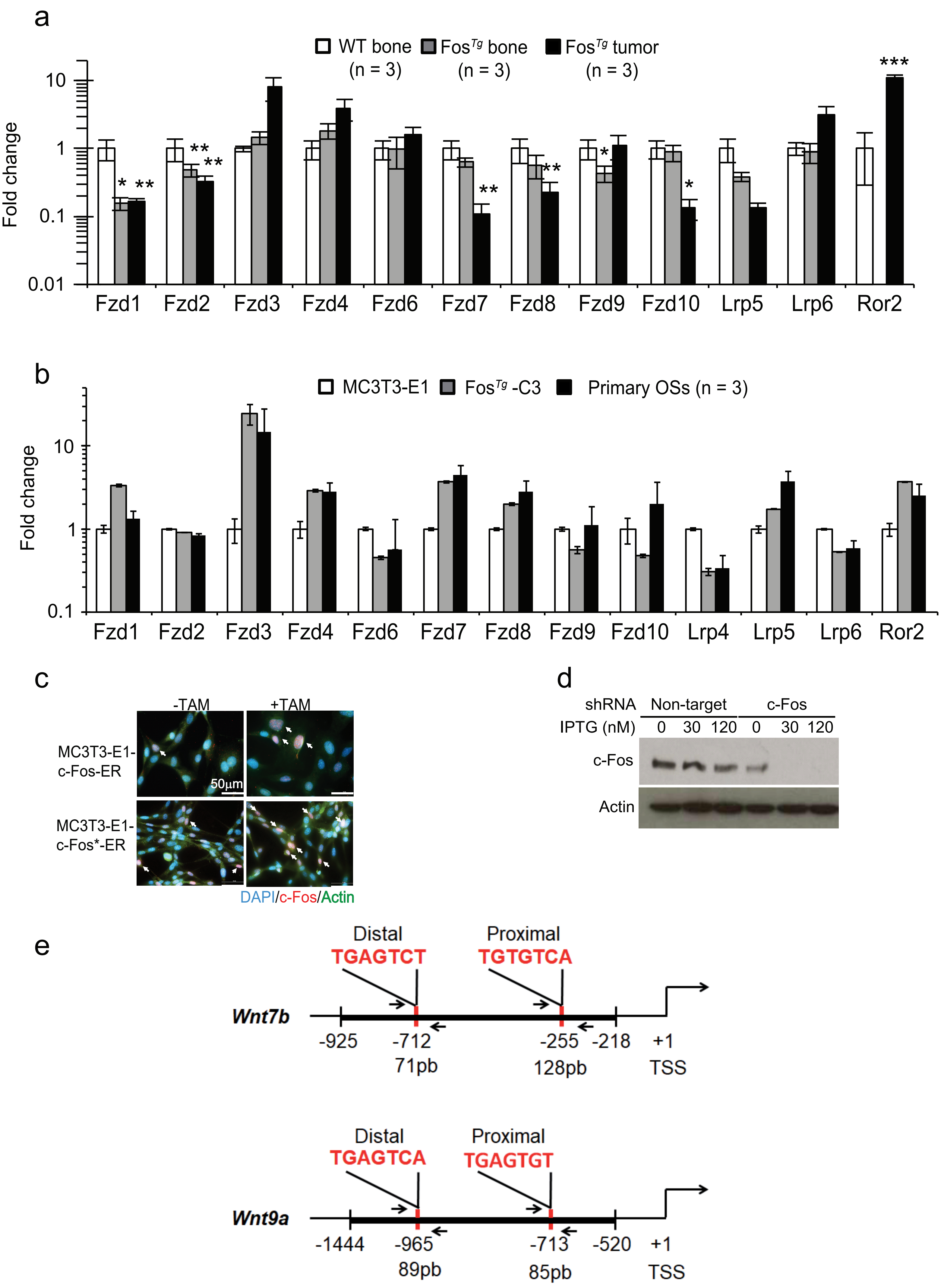

Supplementary information Figure S3. c-Fos/AP-1 promotes the expression of Wnt7b and Wnt9a in OS

qPCR analysis of Wnt receptors and co-receptors in **(a)** H2-*c-fos*LTR bones, tumor-bearing bones and dissected tumors and **(b)** cultivated MC3T3-E1, Fos^{Tg}-C3 and primary OS cells. **(c)** Representative immunofluorescence images of MC3T3-E1-c-Fos-ER and MC3T3-E1-c-Fos*-ER cells stained for c-Fos (red) and actin (green). Nuclei are counterstained with DAPI (blue). **(d)** c-Fos immunobloting in H2-*c-fos*LTR OS cell line (C-3) expressing IPTG-inducible *c-fos* shRNA or non-target shRNA in the presence/absence of IPTG. Actin is used to control protein loading. **(e)** Scheme of the murine *Wnt7b* and *Wnt9a* promoters indicating putative AP-1 binding sites. Bold region represents the genomic fragment cloned in the reporter construct. In D and E bars represent mean ± sem of biological triplicates, except for MC3T3-E1 and Fos^{Tg}-C3 cells where technical triplicates were used. *$P < 0.05$ and ***$P < 0.001$.
