# Supplementary material for: Wnt signaling and Loxl2 promote aggressive osteosarcoma
Source: Cell Res. 2020 Jul 20;30(10):885–901. doi: 10.1038/s41422-020-0370-1 (PMC7608146; doi:10.1038/s41422-020-0370-1)
Supplement: Supplementary file 4 — Supplementary Figure S4 [file 41422_2020_370_MOESM4_ESM.pdf]

Supplementary information Figure S4

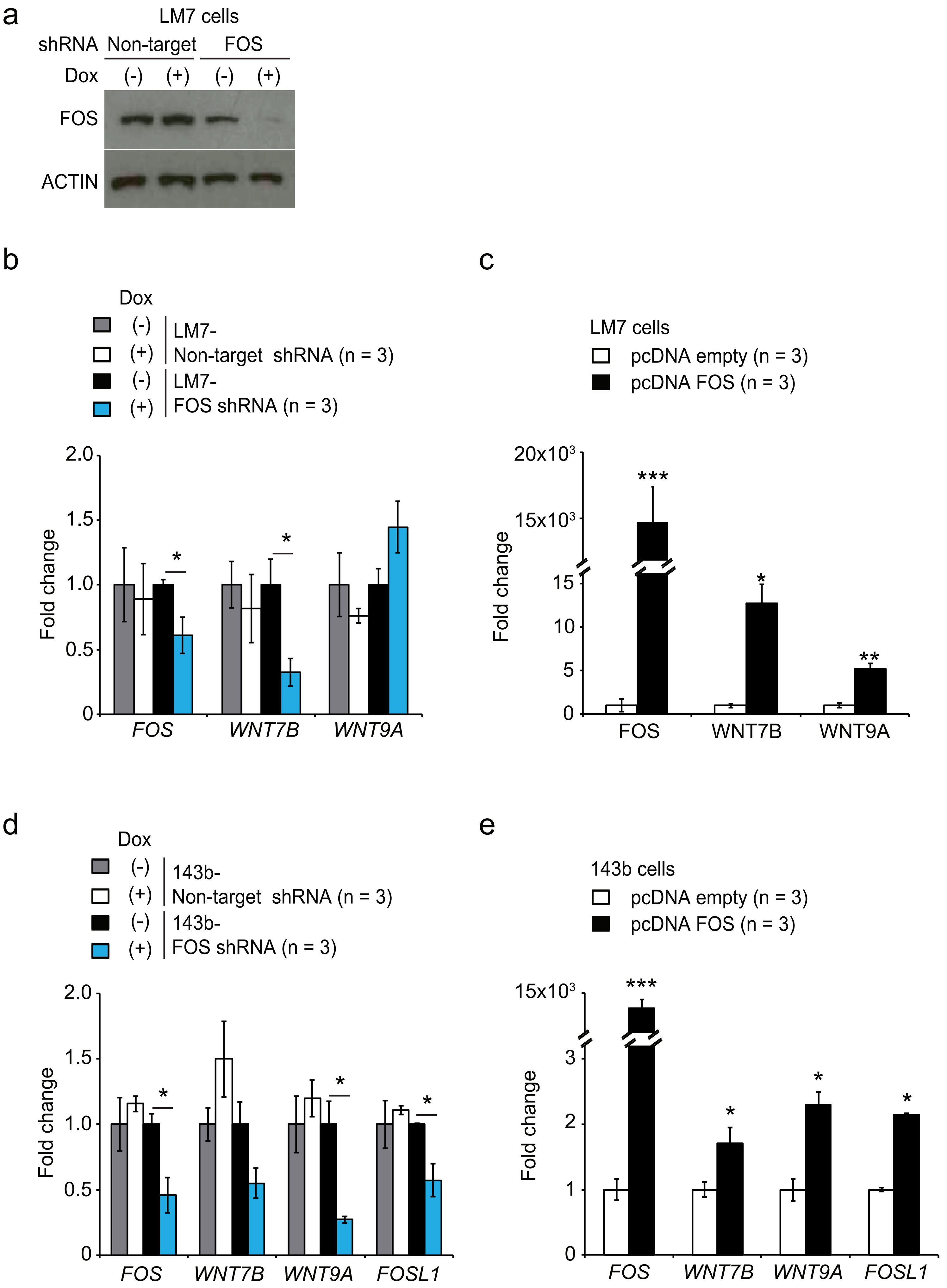

Supplementary information Figure S4. FOS targets WNT7B and WNT9A in a human OS cell line

(a) FOS immunobloting in the LM7 human OS cell line expressing DOX-inducible FOS shRNA or non-target shRNA in the presence/absence of DOX. ACTIN is used to control protein loading. **(b)** Gene expression in the LM7 human OS cell line expressing DOX-inducible *c-FOS* shRNA or non-target shRNA in the presence/absence of DOX was determined by qPCR and the direct c-FOS target gene FOSL1 included as a positive control. **(c)** qPCR analysis of FOS, WNT7B and WNT9A in the LM7 cells 48 hours after ectopic FOS expression. **(d)** Gene expression in the 143b human OS cell line expressing DOX-inducible *c-FOS* shRNA or non-target shRNA in the presence/absence of DOX was determined by qPCR and the direct c-FOS target gene FOSL1 included as a positive control. **(e)** qPCR analysis of FOS, WNT7B, WNT9A and FOSL1 in the 143b cells 48 hours after ectopic FOS expression. Bar represent mean ± sem, respectively. *$P < 0.05$ and ***$P < 0.001$.
