# Supplementary material for: Wnt signaling and Loxl2 promote aggressive osteosarcoma
Source: Cell Res. 2020 Jul 20;30(10):885–901. doi: 10.1038/s41422-020-0370-1 (PMC7608146; doi:10.1038/s41422-020-0370-1)
Supplement: Supplementary file 5 — Supplementary Figure S5 [file 41422_2020_370_MOESM5_ESM.pdf]

Supplementary information Figure S5

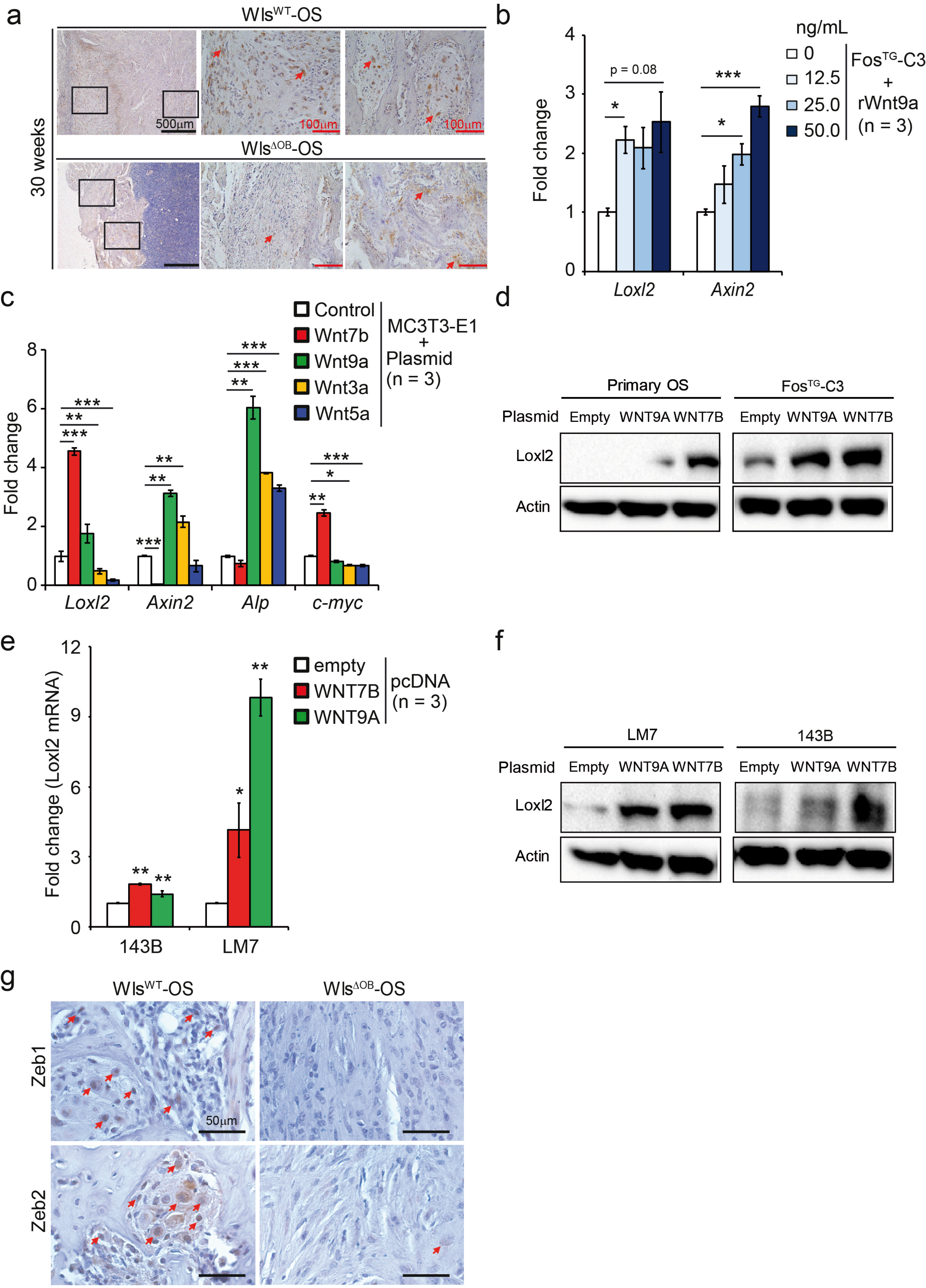

Supplementary information Figure S5. Loxl2 is a Wnt target gene regulating OS growth

(a) IHC analysis of Loxl2 expression in 30 weeks-old Wls^{WT}-OS and $Wls^{\Delta OB}$-OS mice, where Dox was removed at 5 weeks of age. **(b)** qPCR analysis of *Loxl2* and the Wnt target *Axin2* in Fos^{Tg}-C3 cells stimulated 48 hours with increasing concentrations of recombinant mouse Wnt9a. **(c)** qPCR analysis of *Loxl2* and the Wnt targets *Axin2, Alp* and *c-myc* in MC3T3-E1 cells ectopically expressing Wnt7b, Wnt9a, Wnt3a and Wnt5a. **(d)** Immunoblot in primary tumor cells isolated from Wls^{WT}-OS and Fos^{Tg}-C3 cells ectopically expressing Wnt7b and Wnt9a. **(e)** qPCR analysis of LOXL2 in LM7 cells and 143b cells ectopically expressing WNT7B and WNT9A. **(f)** Immunoblot in LM7 cells and 143b cells ectopically expressing WNT7B and WNT9A. **(g)** IHC analysis of Zeb1 and Zeb2 at 15 weeks. Red arrow indicates positive cells. Bar represent mean ± sem, respectively. *$P < 0.05$, **$P < 0.01$ and ***$P < 0.001$.
