# Supplementary material for: Wnt signaling and Loxl2 promote aggressive osteosarcoma
Source: Cell Res. 2020 Jul 20;30(10):885–901. doi: 10.1038/s41422-020-0370-1 (PMC7608146; doi:10.1038/s41422-020-0370-1)
Supplement: Supplementary file 6 — Supplementary Figure S6 [file 41422_2020_370_MOESM6_ESM.pdf]

Supplementary information Figure S6

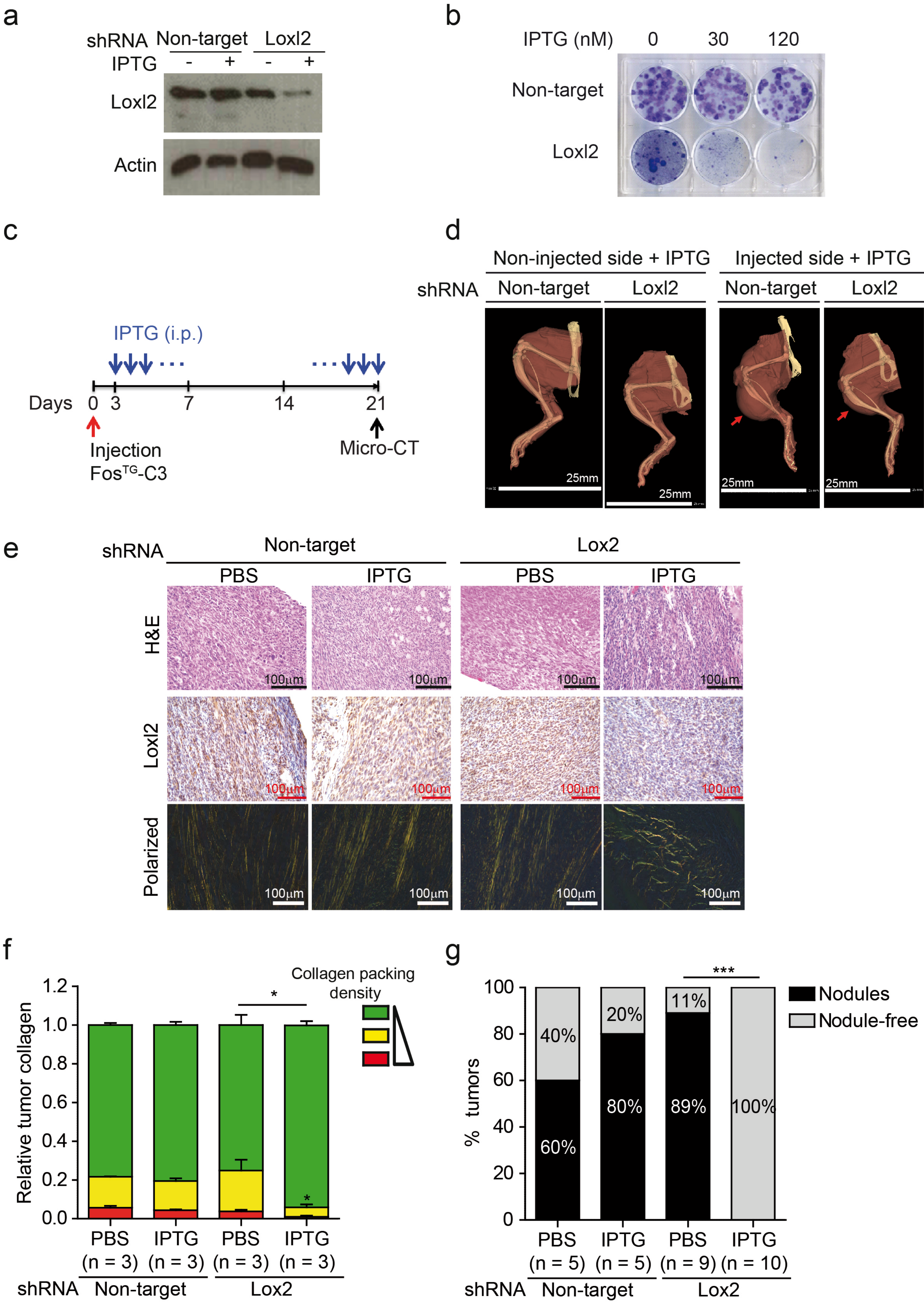

Supplementary information Figure S6. Knock-down of Loxl2 affects tumor collagen structure and suppresses lung colonization

(a) LoxL2 immunoblot in Fos^{Tg}-C3 cells expressing IPTG-inducible *Loxl2* shRNA or non-target shRNA in the presence/absence of IPTG. Actin was used as a loading control. **(b)** Colony formation assay in Fos^{Tg}-C3 cells IPTG-induced Loxl2 shRNA or non-target shRNA. **(c)** Experimental procedure to silence Loxl2 expression in orthotopically xenografted Fos^{Tg}-C3 cells expressing IPTG-induced Loxl2 shRNA or non-target shRNA. Two days post-orthotopic OS cell injection, NSG mice were injected i.p. with PBS or IPTG 5 times/weeks during 3 weeks. **(d)** Representative Micro-CT: 3D reconstructions of orthotopically xenografted Fos^{Tg}-C3 cells at the end point. Red arrow indicates tumors. **(e)** Histological analyses of the tumor expressing IPTG-induced Loxl2 shRNA or non-target shRNA at the end point. Top: H&E, middle: Loxl2 IHC, bottom: picrosirius red. **(f)** Quantification of tumor collagen packing density. Bar graphs represent mean ± sem. *$P < 0.05$ by two-way ANOVA with Bonferroni post-test. **(g)** Incidence of tumor cell lung colonization in the experimental cohorts. ***$P < 0.001$ by Fisher's exact test.
