# Supplementary material for: Wnt signaling and Loxl2 promote aggressive osteosarcoma
Source: Cell Res. 2020 Jul 20;30(10):885–901. doi: 10.1038/s41422-020-0370-1 (PMC7608146; doi:10.1038/s41422-020-0370-1)
Supplement: Supplementary file 7 — Supplementary Figure S7 [file 41422_2020_370_MOESM7_ESM.pdf]

Supplementary information Figure S7

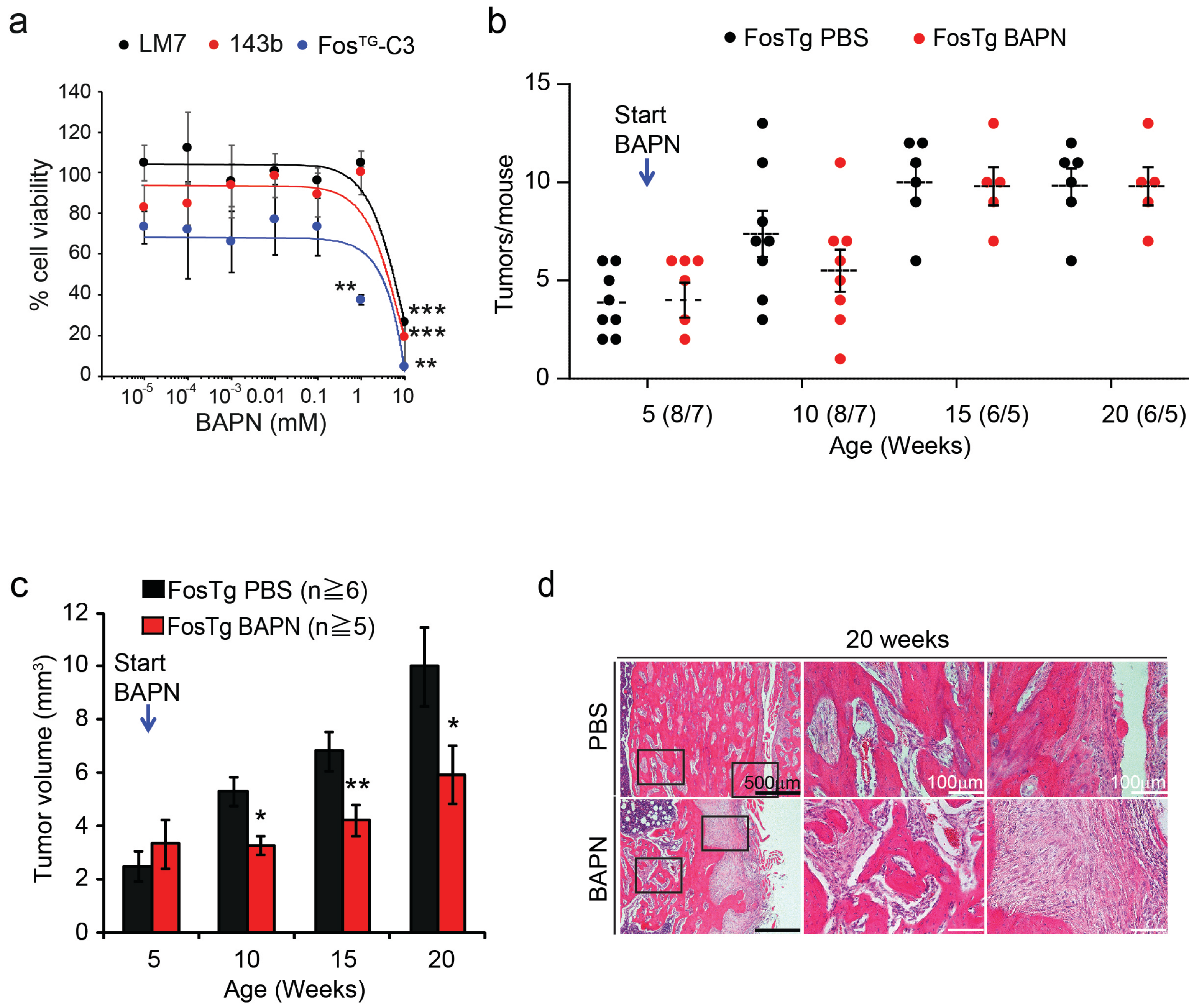

Supplementary information Figure S7. Inhibition of Lysyl oxidase activity reduces OS growth

(a) Mouse and human OS cell lines were cultured with various concentrations of BAPN for 4 days and proliferation quantified by MTT assay. *P < 0.05, **P < 0.01 and ***P < 0.001 by 2-tailed t-test between PBS and BAPN treatment. 5 weeks old H2-*c-fos*LTR mice were injected 3 times/week during 15 weeks with 250 mg/kg BAPN to block lysyl oxidase activity or with vehicle (PBS) and tumors monitored longitudinally by Micro-CT. Tumor number per mouse **(b)** and average tumor volume **(c)** at 5, 15 and 20 weeks. **(d)** Representative tumor histology at end point.
