# Supplementary material for: Wnt signaling and Loxl2 promote aggressive osteosarcoma
Source: Cell Res. 2020 Jul 20;30(10):885–901. doi: 10.1038/s41422-020-0370-1 (PMC7608146; doi:10.1038/s41422-020-0370-1)
Supplement: Supplementary file 8 — Supplementary Figure S8 [file 41422_2020_370_MOESM8_ESM.pdf]

Supplementary information Figure S8

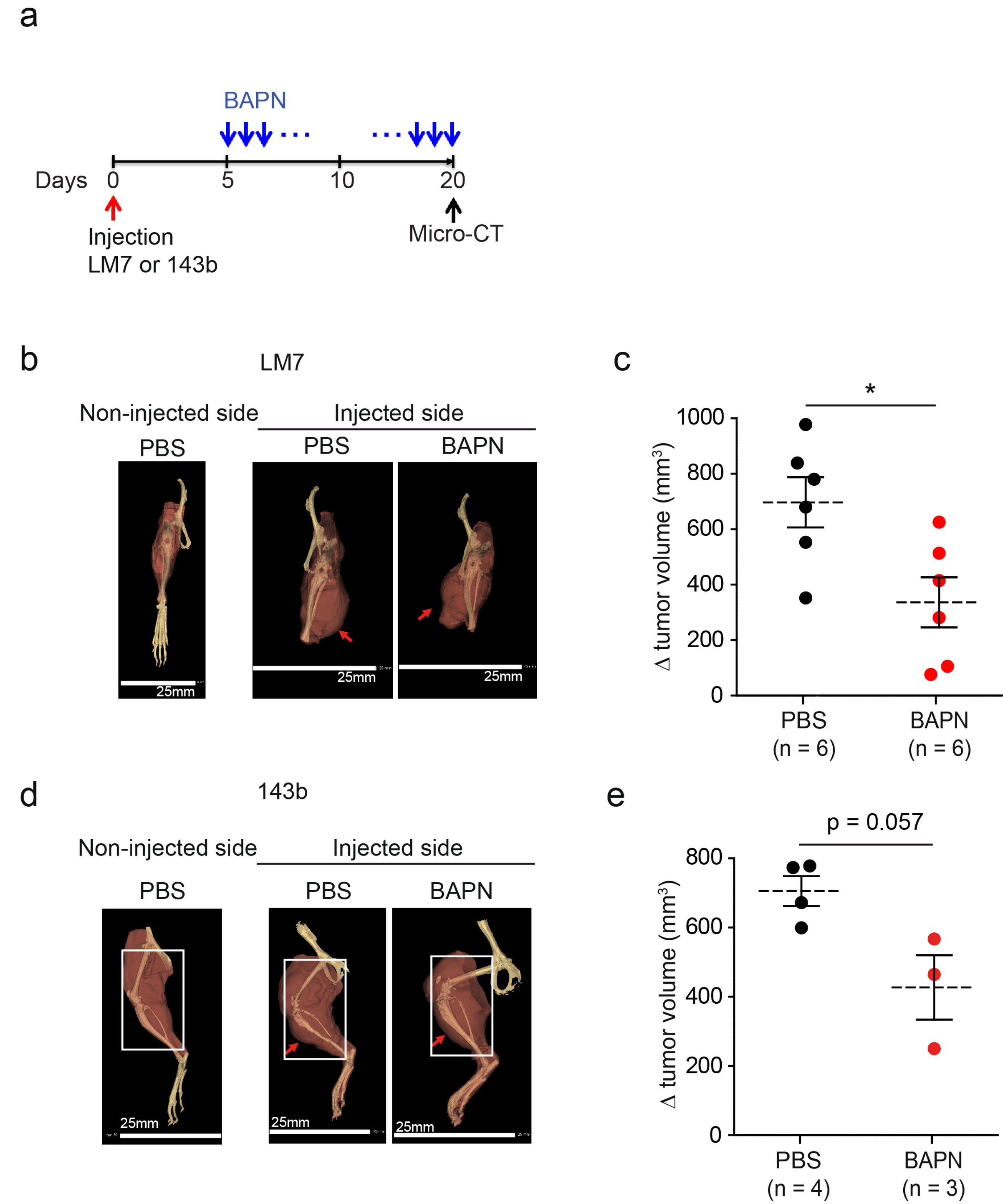

Supplementary information Figure S8. Inhibition of Lysyl oxidase activity reduces OS growth

(a) Experimental procedure to inhibit lysyl oxidases in orthotopically xenografted human OS cell lines LM7 and 143b. Five days post-orthotopic OS cell injection, NSG mice were injected i.p. with PBS or 250 mg/kg BAPN 5 times/week during 15 days **(b-e)** Micro-CT analysis: Representative Micro-CT 3D reconstructions of orthotopically xenografted LM7 **(b)** and 143b **(d)** cells at end point. Red arrow indicates tumors and the white rectangle in **d** indicates the area enlarged in Supplementary information Figure S9d. Average tumor volume at end point for LM7 **(c)** and 143b **(e)** cells. $^{*}P < 0.05$ by two-tailed Mann-Whitney-Wilcoxon test.
