# Supplementary material for: Wnt signaling and Loxl2 promote aggressive osteosarcoma
Source: Cell Res. 2020 Jul 20;30(10):885–901. doi: 10.1038/s41422-020-0370-1 (PMC7608146; doi:10.1038/s41422-020-0370-1)
Supplement: Supplementary file 9 — Supplementary Figure S9 [file 41422_2020_370_MOESM9_ESM.pdf]

Supplementary information Figure S9

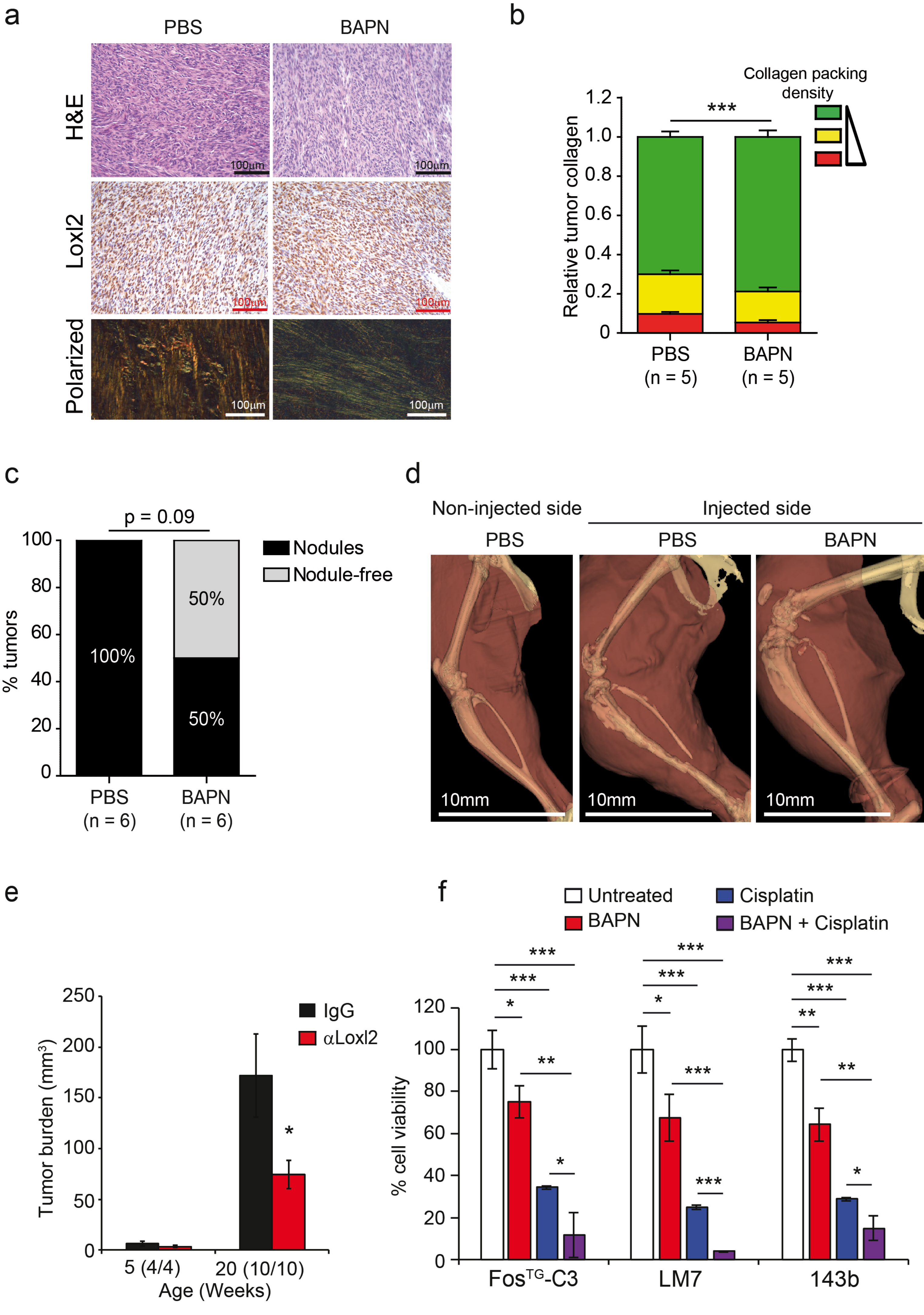

Supplementary information Figure S9. Inhibition of Lysyl oxidase activity or Loxl2 reduces OS growth

(a) Representative images of H&E from LM7 tumor-bearing PBS- and BAPN-treated mice at end point. **(b)** Quantification of tumor collagen packing density in LM7-derived tumors. Bar graphs represent mean ± sem. ***P < 0.001 by two-way ANOVA with Bonferroni post-test. **(c)** Incidence of tumor cell lung colonization in LM7-orthotopic xenografts. Data were analyzed by Fisher's exact test. **(d)** Representative images from 143b-orthotopic xenografts showing tumor-induced bone destruction in tumor-bearing PBS-treated mice (middle panel) compared to non-injected side or injected and BAPN-treated mice. **(e)** Micro-CT quantification of tumor burden at 5 and 20 weeks in H2-*c-fos*LTR mice injected 2 times/week with anti-Loxl2 blocking antibody (30 mg/kg) or with IgG during 15 weeks**.** **(f)** Mouse and human OS cell lines were cultured with 5mM BAPN with or without 100 μM cisplatin for 2 days and proliferation quantified by XTT assay. Bar graphs represent mean ± sem. *P < 0.05 by two-way ANOVA with Bonferroni post-test.
