# Supplementary material for: Wnt signaling and Loxl2 promote aggressive osteosarcoma
Source: Cell Res. 2020 Jul 20;30(10):885–901. doi: 10.1038/s41422-020-0370-1 (PMC7608146; doi:10.1038/s41422-020-0370-1)
Supplement: Supplementary file 10 — Supplementary Figure S10 [file 41422_2020_370_MOESM10_ESM.pdf]

Supplementary information Figure S10

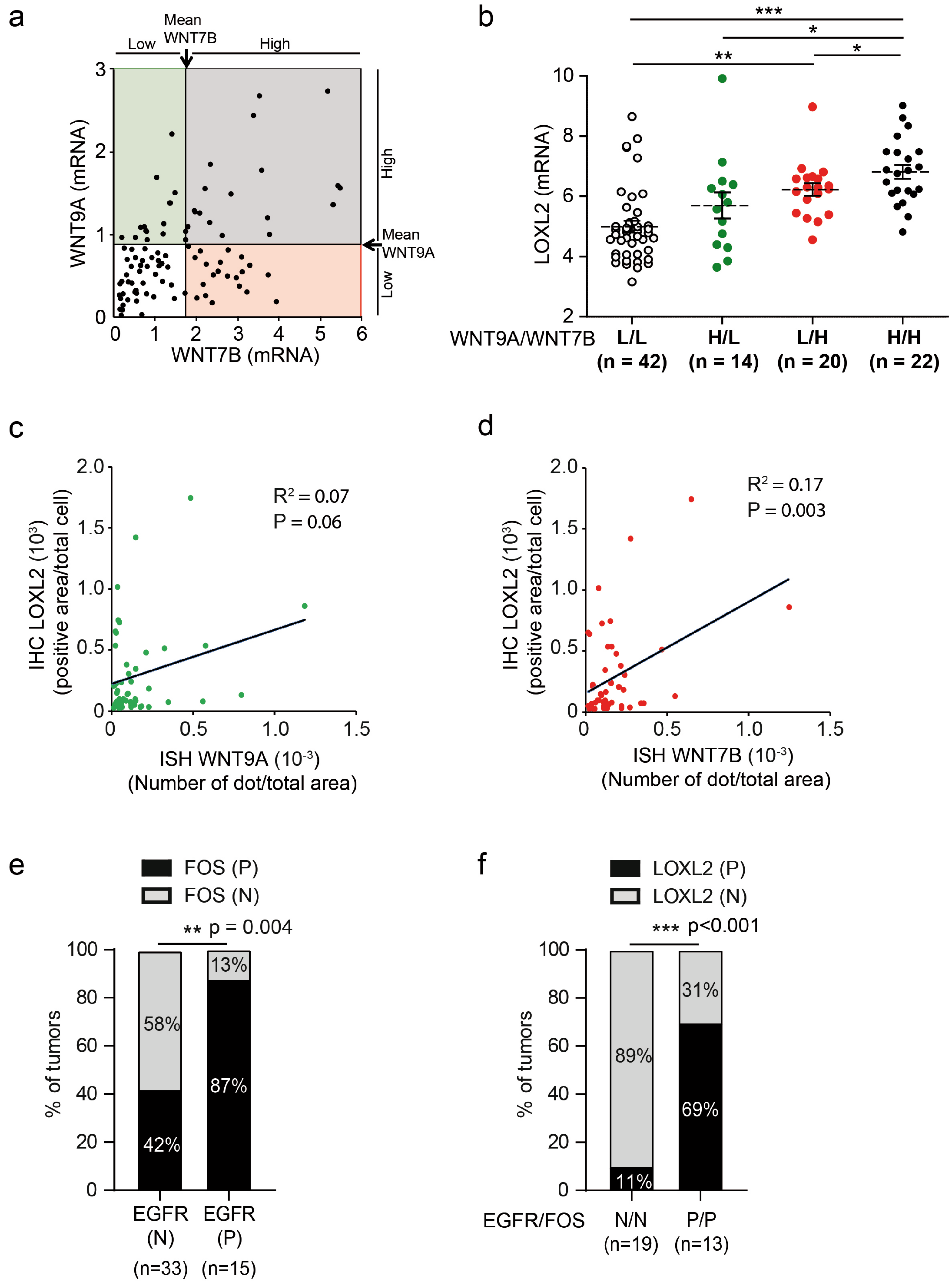

Supplementary information Figure S10. Co-expression of FOS, WNT7B, WNT9A and LOXL2 in human OS

(a) Scatter plot for WNT7B versus WNT9A mRNA expression in human OS (TARGET Data Matrix). RNA-seq values (Transcripts Per Kilobase Million: TPM) are plotted as log2(TPM+1) and divided in 4 groups according to their distribution relative to the mean values for WNT7B and WNT9A: H/H (high WNT9A and high WNT7B; gray), L/H (low WNT9A and high WNT7B; red), H/L (high WNT9A and low WNT7B; green) and L/L (low WNT9A and low WNT7B; white). **(b)** Comparison of LOXL2 expression in the 4 subsets computed from **a.** Plot includes mean ± s.d. *$P < 0.05$, **$P < 0.01$ and ***$P < 0.001$ by unpaired two-tailed *t*-test. Scatter plots of LOXL2 protein expression versus mRNA expression of WNT7B **(c)** and WNT9A **(d)** in human OS TMA (Biomax, n=49). WNT9A and WNT7B mRNA were detected by in situ hybridization (ISH), positivity was scored in a semi-quantitative blinded manner and data plotted against LOXL2 protein expression similarly evaluated by Immunohistochemistry (IHC). Linear regression analysis data (R^2 and P) are indicated in each scatter plot. **(e and f)** Analyses of human OS in US Biomax TMA (OS802c). **(e)** Quantification of EGFR-positive and negative-OS that are either FOS double-positive or – negative in a human OS TMA (n=48). **(f)** Quantification of EGFR/FOS double-positive and negative-OS that are either LOXL2-positive or –negative (n=32).
