# Supplementary material for: Wnt signaling and Loxl2 promote aggressive osteosarcoma
Source: Cell Res. 2020 Jul 20;30(10):885–901. doi: 10.1038/s41422-020-0370-1 (PMC7608146; doi:10.1038/s41422-020-0370-1)
Supplement: Supplementary file 11 — Supplementary Figure S11 [file 41422_2020_370_MOESM11_ESM.pdf]

Supplementary information Figure S11

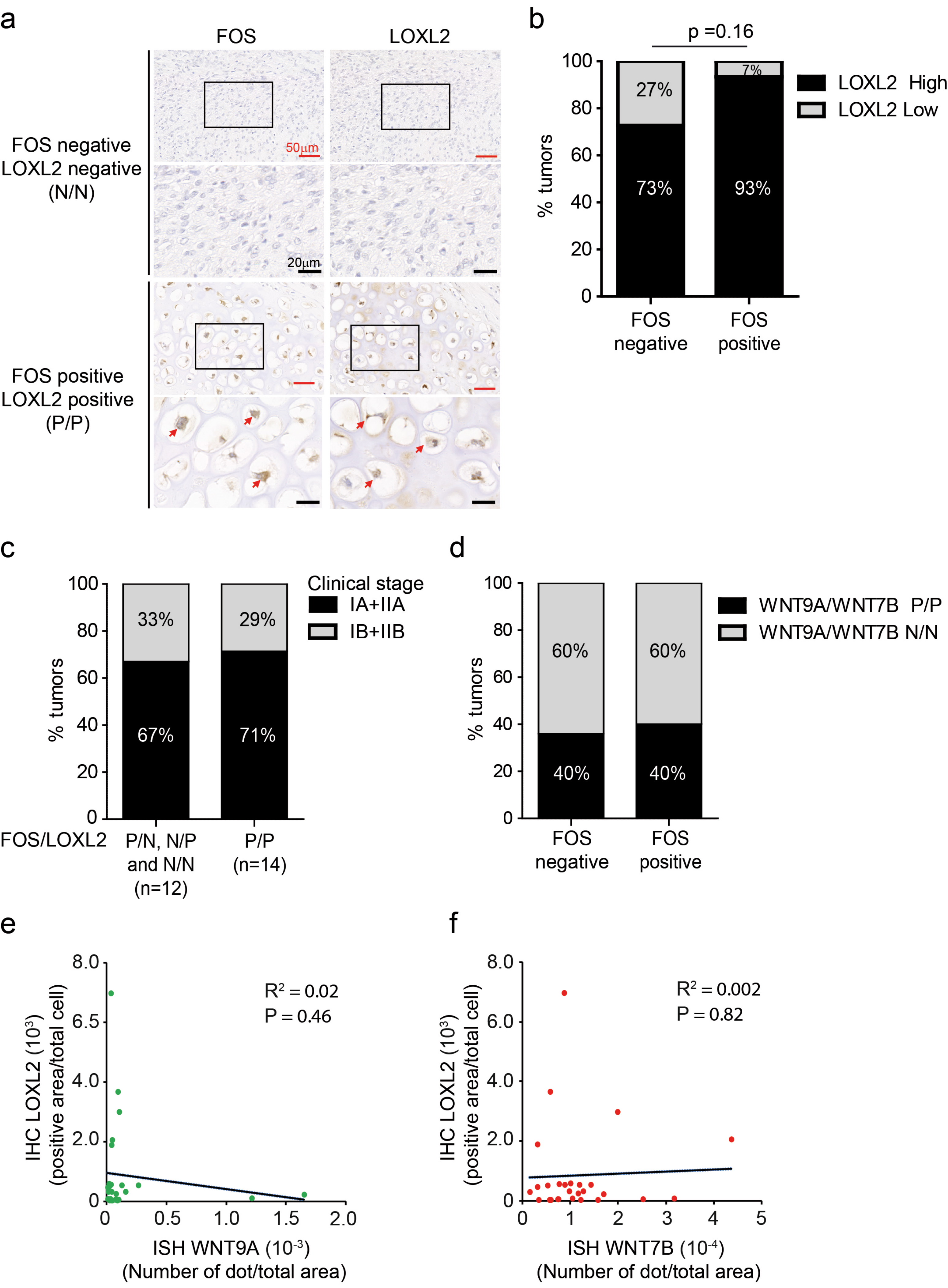

Supplementary information Figure S11. Analyses of human Chondrosarcoma

(a-d) Analyses of human Chondrosarcoma (CS) in US Biomax TMA (OS802c). **(a)** Representative IHC images of FOS/LOXL2 double-negative (N/N) and –double-positive (P/P) human OS. Positive cells are indicated by red arrows. **(b)** Quantification of FOS-positive and -negative CS that are either LOXL2-positive or –negative in a human CS TMA (n=30). **(c)** Correlation of FOS and LOXL2 IHC groups with CS clinical stage. P: positive. N: negative. N/N: double-negative. P/N and N/P: single-positive. P/P: double-positive (n=27). **(d)** Quantification of FOS-positive and -negative CS that are either WNT7B/WNT9A double-positive or –negative in a human CS TMA (n=27). $^*P < 0.05$ and $^{***}P < 0.001$ by Fisher's exact test. Scatter plots of LOXL2 protein expression versus mRNA expression of WNT7B **(e)** and WNT9A **(f)**. WNT9A and WNT7B mRNA were detected by in situ hybridization (ISH), positivity was scored in a semi-quantitative blinded manner and data plotted against LOXL2 protein expression similarly evaluated by Immunohistochemistry (IHC). Linear regression analysis data (R^2 and P) are indicated in each scatter plot (n=27).
