# Supplementary material for: Wnt signaling and Loxl2 promote aggressive osteosarcoma
Source: Cell Res. 2020 Jul 20;30(10):885–901. doi: 10.1038/s41422-020-0370-1 (PMC7608146; doi:10.1038/s41422-020-0370-1)
Supplement: Supplementary file 12 — Supplementary Figure S12 [file 41422_2020_370_MOESM12_ESM.pdf]

Supplementary information Figure S12

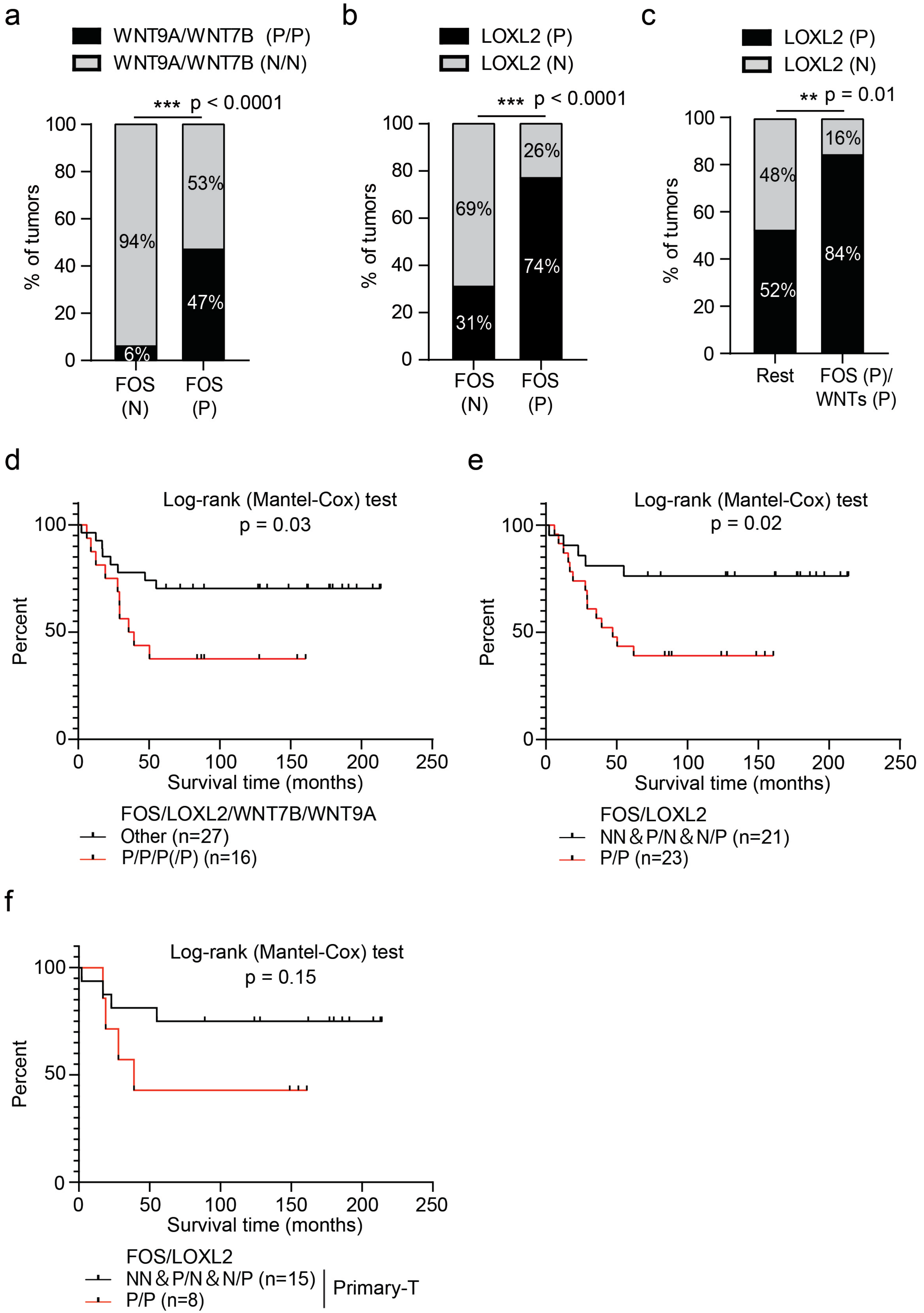

Supplementary information Figure S12. FOS/LOXL2 double positivity is predictive of OS patient survival.

This figure refers to the University Clinic of Navarra TMA (UCN). **(a)** Quantification of FOS-positive and negative-OS that are either WNT7B/WNT9A double-positive or double-negative in a human OS TMA (n=47, indicates core number). **(b)** Quantification of FOS-positive and negative-OS that are either LOXL2-positive or -negative (n=67, indicates core number). **(c)** Quantification of FOS/WNT (WNT7B, WNT9A and WNT7B/WNT9A) double and triple positive and negative-OS that are either LOXL2-positive or -negative (n=58, indicates tissue spot number)**.** P: positive. N: negative. N/N: double-negative. P/N and N/P: single positive. P/P: double-positive. ****P* < 0.001 by Fisher's exact test. **(d, e** and **f)** Overall survival of OS patients according to FOS/LOXL2/WNT (WNT7 and/or WNT9A) -positivity and FOS/LOXL2 double-positivity. **(d** and **e)** All patients (primary tumor, post-treatment, metastatic and recurrent osteosarcoma) are included **(f)** Only patients with primary tumors before chemotherapy are included in the analysis. Data are analyzed by Log-rank test and patient number is indicated.
