# Supplementary material for: Wnt signaling and Loxl2 promote aggressive osteosarcoma
Source: Cell Res. 2020 Jul 20;30(10):885–901. doi: 10.1038/s41422-020-0370-1 (PMC7608146; doi:10.1038/s41422-020-0370-1)
Supplement: Supplementary file 13 — Supplementary Table S1 [file 41422_2020_370_MOESM13_ESM.pdf]

Supplementary information, Table S1. Primers and Plasmids

Oligonucleotide primers for quantitative PCR – mouse genes:

Gene	Forward primer (5'-3')	Reverse primer (5'-3')
Wls	ATCCGTGACATCGAGGAAGC	GGAACCATGGACTCATCTCCA
Axin2	GACCGACGATTCCATGTCCA	GCGGTGGGTTCTCGGAAAAT
c-myc	TCACCAGCACAACTACGCCG	TGCTTCAGGACCCTGCCACT
Ccnd1	TGCTGCAAATGGAACTGCTT	GGTCTGCTTGTTCTCATCCG
Cre	CAATTTACTGACCGTACAC	TAATCGCCATCTTCCAGCAG
c-fos (Endogenous)	ATGGTGAAGACCGTGTCAGG	GTTGATCTGTCTCCGCTTGGA
c-fos (Transgene)	TGTGTTCCTGGCAATAGCGTGT	GGCAATTCCGCCCATAGTGA
Runx2	GATGCGTATTCCTGTAGATCCG	GCTGTTGTTGCTGTTGCTGT
Sp7	ATGGCGTCCTCTCTGCTTG	TGAAAGGTCAGCGTATGGCTT
Alp	TCTGGTGGCATCTCGTTATC	CCTGAAACTCCAAAAGCTC
Bglap	ACCCTGGCTGCGCTCTGTCTCT	GATGCGTTTGTAGGCGGTCTTCA
Sost	TGAGAACAACCAGACCATGAA	CAGCTGTACTCGGACACAT
Wnt7b	CTGAGCAATTGTGGCTGTGA	CATCCACAAAGCGACGAGAA
Wnt9A	TCGTGGGTGTGAAGGTGATA	GTTTTAGGTGCTTGCCCACC
Wnt3a	CTCGGATACCTCTTAGTGCTC	TGCTCAGAGAGGAGTACTGG
Fosl1	AGAGCTGCAGAAGCAGAAGG	CAAGTACGGGTCCTGGAGAA
Lox	CCCGACCCCTACTACATCCA	ACTGGCCAGGCAGTTTTCTT
Loxl2	ATGAAGTCCAGTGCACAGGG	CCCGGCATCTTCTTCATGGT
Loxl3	CTTCACAGAAGCCACAGGGT	CACACTTCCTTCAGTCCCCC
Loxl4	CCCAGCTGGTACAAGAGACG	CGGACTTGGAAAGGCAGTTT
Plod2	GCCATGGAAGCATAGCCACT	GAGACTTGCCCTGGAGGAAC
P4ha2	GCTGTCTTCCAACTGGGTGA	GTACCGCAGATTCCCTCCAG

Oligonucleotide primers for quantitative PCR – human genes:

Gene	Forward primer (5'-3')		Reverse primer (5'-3')
FOS	AGACAGCCCGCTCCGTGCCAG AC		CGGGGGTAGGTGAAGACGAAGGAAG AC
WNT7 B	GGCTACTACAACCAAGCCGA		TTCTTGATCTCCCGAGCGTC
WNT9 A	TCGAGTGCCAGTTCCAGTTC		AAGGCAGTCTCCTTGAAGCC
LOXL2	GCCACATAGGTGGTTCCTTCA		GAAGATGTGGTGTGGCCTGA

Oligonucleotide primers for ChIP-quantitative PCR:

Fosl1	CCACGAAGTGTTGGGATGT	TCGACAGGAAATGGACACC
Wnt7b (Distal)	CTTTTAATGCTGGGCAGGCT	CCGGATCAGGCATTGTTACC
Wnt7b (Proximal)	AGCGGGTAGTTTCTTAGCCC	CAAAGAGACACCTGCGCTC
Wnt9a (Distal)	AGCTCAAAACCCTCAGACTT	GAAGGCAAACCTCCTTACCA
Wnt9a (Proximal)	TAAGCAGTCTATTGGGTGCC	TGTGACTCATCTTCGTGGAC

Plasmids:

Human

pcDNA-WNT7B: Addgene, #35915 (constructed by Dr. Marian Waterman[79])
pcDNA-WNT9A: Addgene, #35918 (constructed by Dr. Marian Waterman[79])
pcDNA3-FOS, constructed by Dr. Bakiri Latifa[80]

Mouse:

pLNC-Wnt3a-HA: Addgene, #18030 (constructed by Dr. Jan Kitajewski[78])
pLNC-Wnt5a-HA: Addgene, #18032 (constructed by Dr. Jan Kitajewski[78])
pLNC-Wnt7b-HA: Addgene, #18037 (constructed by Dr. Jan Kitajewski[78]).
pCAGG Wnt9a-HA: Constructed by the lab of Dr. Christine Hartmann
pBabe-c-fos-ER and pBabe-c-fos-ER*, PMID: 10655067
pCDNA-c-Fos, constructed by Dr. Bakiri Latifa[81]

Luciferase reporters

pGL4.23[luc2/minP] empty reporter: Promega. Cat. No. E8411
pGL4.23[luc2/minP]-Wnt7b reporter: constructed by Dr. Latifa Bakiri.
pGL4.23[luc2/minP]-Wnt9a reporter: constructed by Dr. Latifa Bakiri.
pGL4.23[luc2/minP]-Fosl1 reporter : Gift from Dr. Meinrad Busslinger[83].
phRG-Renilla: Promega. Cat. No. E2241

Lentiviral shRNA expression vectors:

pVSV-G: Addgene, #8454
pCMV delta R8.2: Addgene, #12263
pLKO-puro-IPTG-3xLacO empty vector: Sigma, #SHC332V
Tet-pLKO-neo empty vector: Addgene, #21916[82]
