# Supplementary material for: Wnt signaling and Loxl2 promote aggressive osteosarcoma
Source: Cell Res. 2020 Jul 20;30(10):885–901. doi: 10.1038/s41422-020-0370-1 (PMC7608146; doi:10.1038/s41422-020-0370-1)
Supplement: Supplementary file 14 — Supplementary Table S2 [file 41422_2020_370_MOESM14_ESM.pdf]

Supplementary information Table S2, Antibody/Antigen retrieval methods

Name	Company (catalog no.)	Application	Conditions
c-Fos (9F6)	Cell Signaling Technology (#2250)	WB, ChIP	WB, 1:1000 dilution<br>ChIP, 1μg antibody/10μl dynabeads
c-Fos (4)	Santa Cruz Biotechnology (sc-52)	IHC	IHC, 1:200 dilution<br>Antigen retrieval: Unitrieve, 60°C for 1 hour
Loxl2	Abcam (ab96233)<br><br><br><br>Novus Biologicals (NBP2-75559)	WB, IHC<br><br><br><br>WB	WB, 1:2000 dilution<br>IHC, 1μg/ml<br>Antigen retrieval: Citrate buffer (pH 6.0) in a pressure cooker or a microwave<br>WB, 1:1000 dilution
Osteocalcin	Enzo laboratories (ALX-210-333)	IHC	IHC, 2 μg/ml<br>Antigen retrieval: Cerezyme I Trypsin Kit, room temperature, 20 min
Sclerostin	R&D Systems (AF1589)	IHC	IHC, 10 μg/ml<br>Antigen retrieval: Cerezyme I Trypsin Kit, at R.T. for 20 min or Unitrieve, 60°C 1 hour
WNT7B	Sigma (SAB2701193)	IHC	IHC, 1:100 dilution<br>Antigen retrieval: Cerezyme I Trypsin Kit, room temperature, 20 min
WNT9A	Abcam (ab125957)	IHC	IHC, 1:100 dilution<br>Antigen retrieval: 20μg/ml Proteinase K at R.T. for 20 min
Cre recombinase	BioLegend (908001)	IHC	IHC, 1:500 dilution<br>Antigen retrieval: Citrate buffer (pH 6.0), in a pressure cooker
Actin	Sigma (A2228)	WB	WB, 1:2000 dilution
Zeb1	Sigma (SAB3500514)<br>(HPA027524)	WB<br><br>IHC	WB, 1:1000 dilution<br><br>IHC, 1:125 dilution<br>Antigen retrieval: Citrate buffer (pH 6.0) in a microwave.
Zeb2	Santa Cruz Biotechnology (sc-48789)	WB, IHC	WB, 1:250 dilution<br>IHC, 1:50 dilution<br>Antigen retrieval: Tris-EDTA buffer (pH 9.0) in a microwave.
